# FIP200 regulates plasma B cell differentiation via mitochondrial and heme homeostasis

**DOI:** 10.1084/jem.20250535

**Published:** 2025-12-17

**Authors:** Liling Xu, Maria Bottermann, Paula M. Villavicencio, John Warner, Stephanie R. Weldon, Zhenfei Xie, Andrew Filby, Xiaotie Liu, Ian G. Ganley, Alison E. Ringel, Usha Nair, Facundo D. Batista

**Affiliations:** 1 Batista Lab, The Ragon Institute of Mass General Brigham, MIT, and Harvard, Cambridge, MA, USA; 2 Institute of Cellular Medicine, Newcastle University, Newcastle upon Tyne, UK; 3 Medical Research Council Protein Phosphorylation and Ubiquitylation Unit, School of Life Sciences, University of Dundee, Dundee, UK; 4 Ringel Lab, The Ragon Institute of Mass General Brigham, MIT, and Harvard, Cambridge, MA, USA; 5 Biology Department, Massachusetts Institute of Technology, Cambridge, MA, USA; 6 Department of Immunology, Harvard Medical School, Boston, MA, USA

## Abstract

Little is known about the role of autophagy in the human humoral immune system. Here, we found that in B cells, genetic ablation of FIP200, a mammalian metabolic sensor that regulates autophagy in response to a range of stimuli, led to diminished humoral immune responses in mice. FIP200-deficient B cells displayed decreased differentiation into plasma cells, as well as mitochondrial dysfunction, alterations in heme biosynthesis, and significant cell death. Notably, the addition of heme was sufficient to rescue plasma cell differentiation of FIP200-deficient B cells. Thus, FIP200 determines B cell fates by controlling mitophagy and metabolic reprogramming.

## Teaser

Xu et al. demonstrate that FIP200, a sensor involved in autophagy regulation, governs the differentiation of plasma B cells. B cells without FIP200 are dysfunctional in mitophagy and metabolism. Adding heme can rescue this phenotype and allow plasma cell differentiation.

## Graphical Abstract

**Figure:**
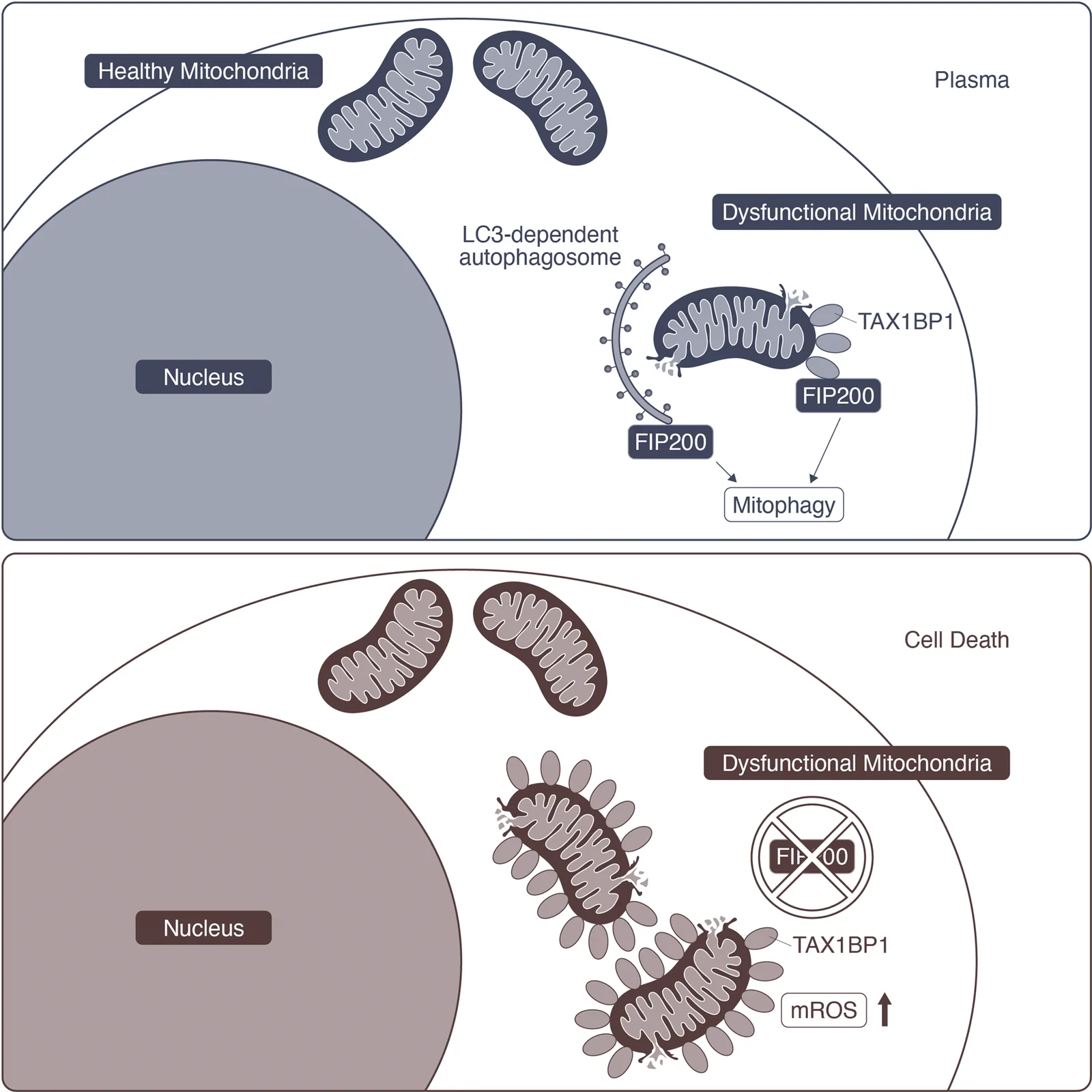

## Introduction

B cell differentiation is a tightly regulated process of cell proliferation, selection, and death, involving multiple interactions with other immune cells. B cells express diverse B cell receptors (BCRs) used to recognize antigens presented by follicular dendritic cells or subcapsular sinus macrophages located in the spleen or lymph nodes (Batista and Harwood, 2009; Carrasco and Batista, 2007; Junt et al., 2007; Phan et al., 2007). After undergoing activation by a cognate antigen, B cells proliferate rapidly, leading to the development of germinal centers (GCs), where they compete for T cell help and may differentiate into memory B cells or plasma cells (Rajewsky, 1996; Victora and Nussenzweig, 2012). Successful humoral immune responses therefore require that activated B cells undergo a metabolic shift marked by enhanced glucose uptake and a concomitant increase in glycolysis, as well as increased mitochondrial mass (Jellusova et al., 2017; Martinez-Martin et al., 2017). Recent work has highlighted the criticality of mitochondrial transcription, translation, and metabolites to activated B cells entering and maintaining GC responses (Haniuda et al., 2020; Yazicioglu et al., 2023; Urbanczyk et al., 2022; Tsui et al., 2018; Jellusova et al., 2017), and mitochondrial homeostasis is a determinant of activated B cell fate *in vitro* (Jang et al., 2015). One mechanism by which mitochondrial homeostasis is maintained is autophagy.

Autophagy, and specifically the crosstalk between autophagy and mitophagy, has been identified as a major factor in the survival of B1 B cells (Clarke et al., 2018) and memory B cells (Chen et al., 2014, 2015; Martinez-Martin et al., 2017), and the survival of plasma cells (Conway et al., 2013; Pengo et al., 2013). B1a cells lacking autophagy show increased mitochondrial mass and mitochondrial membrane potential, which result in self-renewal defects (Clarke et al., 2018). Memory B cells deficient in autophagy accumulate dysfunctional mitochondria with higher mitochondrial reactive oxygen species (mROS), resulting in oxidative stress and, ultimately, cell death (Chen et al., 2014). While the link to mitochondrial function has not been directly observed in plasma cells deficient in autophagy, ATP production after LPS stimulation significantly decreases in those cells (Pengo et al., 2013). The biology of the GC is particularly dependent on autophagy: during viral infections, rates of autophagy are highest in those B cells resident in the GC, and mitochondrial and metabolic homeostasis in GC B cells is highly dependent on WD-repeat domain, phosphoinositide–interacting protein 2 (WIPI2), which mediates LC3 lipidation, autophagosomal membrane formation, and the balance between canonical and noncanonical autophagy pathways (Dooley et al., 2014; Martinez-Martin et al., 2017). Above all, autophagy plays an important role in regulating mitochondrial homeostasis in B cells at a number of different stages and is essential for maintaining memory B and long-lived plasma cells.

Previous studies on autophagy in B cells focused on the downstream end of the autophagy pathway, related to the lipidation of LC3 (Chen et al., 2014; Martinez-Martin et al., 2017; Pengo et al., 2013). Focal adhesion kinase family–interacting protein of 200 kDa (FIP200) is one of the initiators in autophagy pathway; it forms a complex with Unc-51-like kinase 1 (ULK1), ATG13, and ATG101, and is essential for the stability and proper phosphorylation of ULK1 and nucleation of the phagophore (Dikic and Elazar, 2018; Hara et al., 2008; Liang et al., 2010). Sequestosome 1 (SQSTM1)/p62 and nonlipidated LC3 accumulate in FIP200-deficient nonimmune and immune cells, indicating suppression of the autophagy pathway (Hara et al., 2008; Wang et al., 2013; Wei et al., 2011; Xia et al., 2017). Furthermore, studies have demonstrated that under certain conditions, FIP200 promotes autophagosome formation through an LC3-independent pathway, interacting with the N-terminal SKIP carboxyl homology (SKICH) domain of NDP52 and TAX1BP1, one of the SQSTM1-like family of autophagy receptors, to form autophagosomes and promote the degradation of aggregated ubiquitinated clusters (Huyghe et al., 2022; Ohnstad et al., 2020; Schlütermann et al., 2021; Vargas et al., 2019). Beyond its role in autophagy, FIP200 has proved to be a versatile molecule: FIP200 interacts with the TSC1-TSC2 complex to regulate cell size (Gan et al., 2005); in ovarian cancer patients, translationally inhibiting FIP200 expression drives high apoptosis in T cells (Xia et al., 2017). However, the role of FIP200 in B cell development and differentiation remains elusive.

Here, we have studied the function and mechanism of FIP200 in B cell differentiation and antibody production. We found that FIP200 acted to regulate plasma cell differentiation and memory response after antigen exposure. Furthermore, the loss of FIP200 contributed to increased mROS, impaired heme metabolism, and the accumulation of mitochondrial mass, ultimately affecting B cell-fate decisions. Our work demonstrates a key role of FIP200 in regulating plasma differentiation through complex interactions between autophagy, metabolism, and mitochondria.

## Results

### Loss of FIP200 impairs plasma cell formation and humoral responses

**Figure S1.**
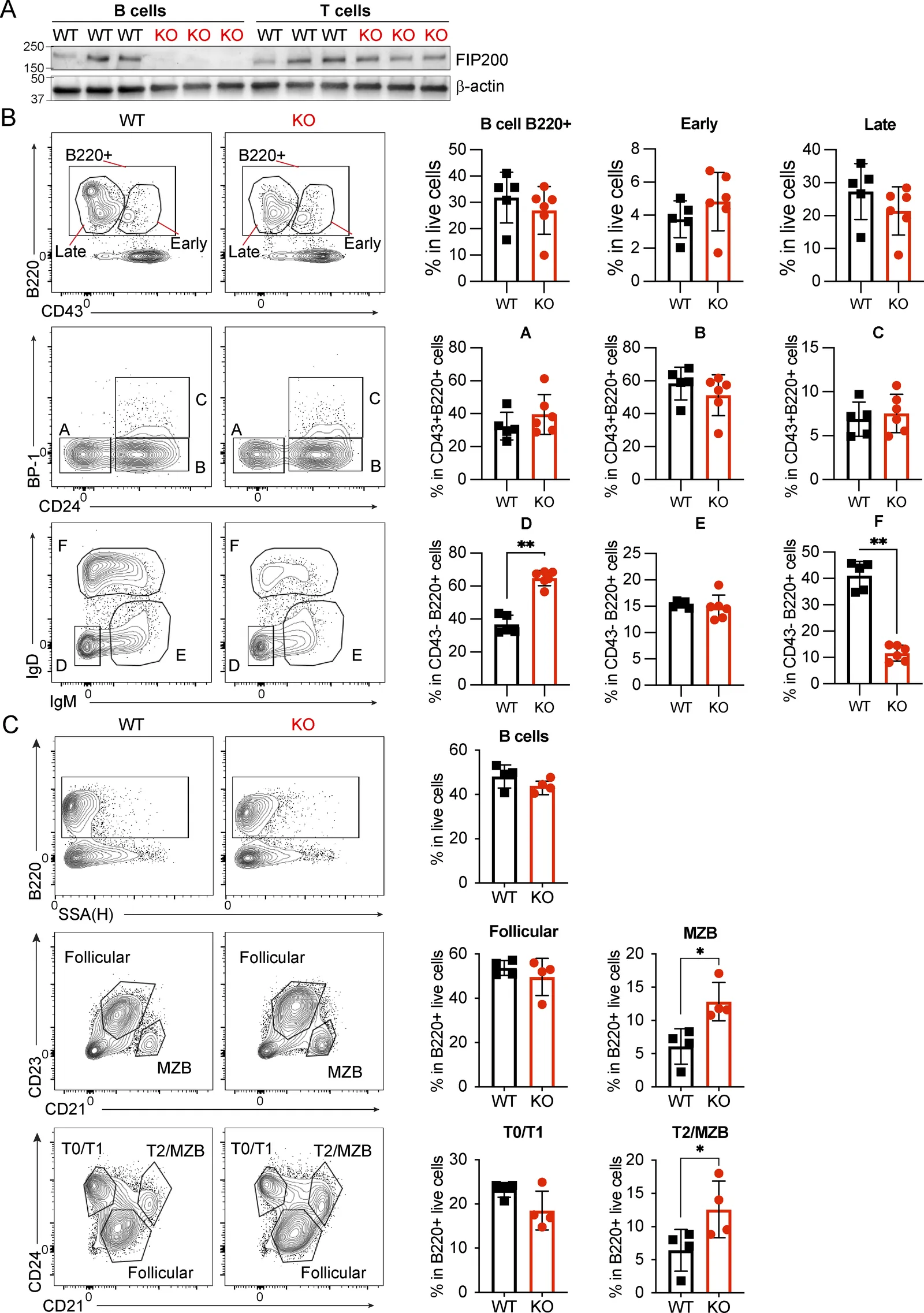
**B cell development in B-*Fip200*** ^***−/−***^ **mice, related to Fig. 1. (A)** Naïve B cells and CD4^+^ T cells were isolated from the spleens of WT or B-*Fip200*^*−/−*^ mice (*n* = 3), and FIP200 expression was detected by western blot. **(B and C)** Flow cytometry analysis of BM (B) or spleen (C) from WT and *Fip200*-KO-B mice. **(B)** BM samples were stained with antibodies against CD43, CD24, BP-1, IgM, IgD, and B220, and populations were identified following the Hardy classification system: B cells (B220^+^), early progenitors (B220^+^CD43^+^), late progenitors (B220^+^CD43^−^), fraction A (CD43^+^CD24^−^BP-1^−^), fraction B (CD43^+^CD24^+^BP-1^−^), fraction C (CD43^+^ CD24^+^ BP-1^+^), fraction D (CD43^−^IgM^−^IgD^−^), fraction E (CD43^−^IgM^+^IgD^−^), fraction F (CD43^−^IgM^+^IgD^+^). Left to right: **P = 0.0043, **P = 0.0043. **(C)** Spleen samples were stained with antibodies against CD21, CD23, CD24, and B220, and transitional or mature B cell populations were identified—B cells (B220^+^), T0-T1 cells (B220^+^CD21^lo^CD24^hi^), T2-MZB cells (B220^+^CD21^hi^CD24^hi^), MZB cells (B220^+^CD21^hi^CD23^lo^), follicular B cells (B220^+^CD21^hi^CD23^+^). Two replicates were performed with three to five animals in each group; one representative experiment is shown. Significant P values were determined by an unpaired *t* test. Top to bottom: *P = 0.0286, *P = 0.0286. Source data are available for this figure: SourceData FS1.

Although downstream components of the autophagy pathway have emerged as important regulators of B cell activation, differentiation, and maintenance (Chen et al., 2014, 2015; Martinez-Martin et al., 2017; Pengo et al., 2013), the role of upstream autophagy effectors such as FIP200 is not clear. Here, we crossed *Fip200*^*flox*/*flox*^ (Liang et al., 2010) mice with *Mb1*-Cre mice (B6.C(Cg)-*Cd79a*^*tm1(cre)Reth*^/EhobJ) (Hobeika, 2006) to generate B cell–specific *Fip200*^−/−^ mice (B-*Fip200*^*-/-*^); *Fip200*^*flox/flox*^*Mb1*-Cre^−/−^ siblings served as controls (WT). To confirm the deletion only occurred in B cells, we isolated naïve B cells and CD4^+^ T cells from the spleens of B-*Fip200*^−/−^ mice: FIP200 protein expression was detected by western blot in CD4^+^ T cells, but not in B cells (Fig. S1 A). We then characterized B cell development in the bone marrow (BM) and spleens of B-*Fip200*^−/−^ by flow cytometry, applying the Hardy classifications (Hardy et al., 1991). Early (CD43^+^) B cell progenitors in the BM were identical between WT and B-*Fip200*^−/−^; however, there were fewer mature B cells in B-*Fip200*^−/−^ overall, but an overabundance at the small pre-B stage (Fig. S1 B; fraction D [CD43^−^IgM^−^IgD^−^]). Nonetheless, splenic B cell populations were comparable between B-*Fip200*^−/−^ and WT, and B-*Fip200*^−/−^ B cell precursors displayed an unimpeded capacity for maturation in the periphery: there was an increase in marginal zone B cells (defined as B220^+^CD21^hi^CD24^hi^CD23^−^) in the spleen relative to controls, but similar numbers of mature follicular (B220^+^CD21^hi^CD24^lo^) B cells (Fig. S1 C). Overall, these results suggest that FIP200 is important for late pre-B cells transitioning to immature and mature B cell stages in the BM, but dispensable for B cell maturation in the spleen.

**Figure 1.**
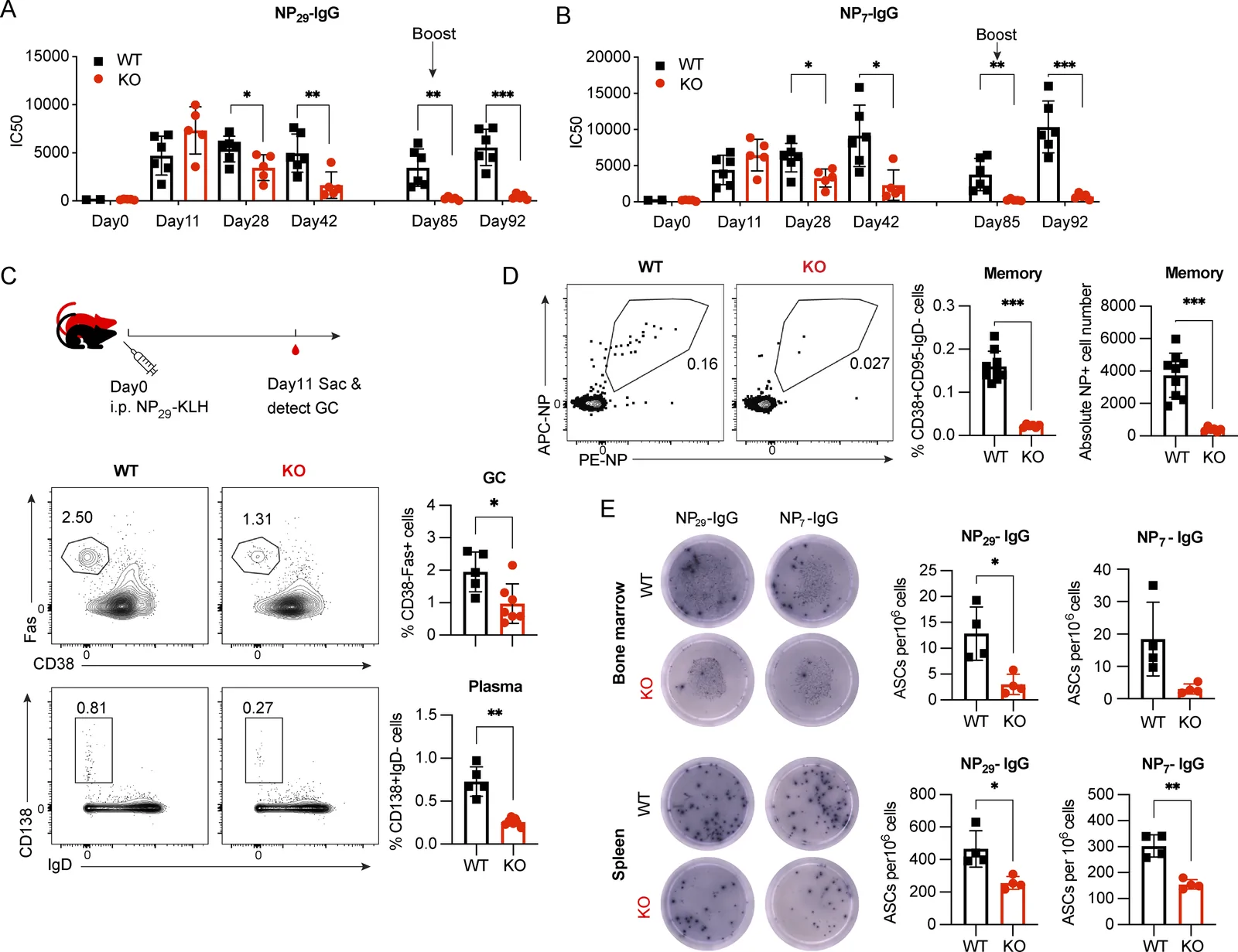
**B cell–specific FIP200-deficient mice showed an impaired humoral immune response. (A and B)** Six WT and five B-*Fip200*^*−/−*^ mice were immunized with 30 μg NP_29_-KLH with Imject Alum and boosted at day 85 with 30 μg NP_29_-KLH in PBS. Mouse serum was collected throughout, and IC50 of anti-NP_29_ (A)– or anti-NP_7_ (B)–specific IgG antibodies was measured by ELISA. Serum collection days are marked on the x axes. **(A)** Left to right: *P = 0.0420, **P = 0.0098, **P = 0.0096, ***P = 0.0010 (unpaired *t* test). **(B)** Left to right; *P = 0.023, *P = 0.0102, **P = 0.0070, ***P = 0.0002 (unpaired *t* test). **(C)** Top: WT (*n* = 5) and B-*Fip200*^*−/−*^ (*n* = 3–7) mice were immunized with 50 μg NP_29_-KLH with Imject Alum and sacrificed at day 11. Bottom: Representative flow cytometry plots and frequency of Fas^+^CD38^−^ GC and CD138^+^ plasma B cells from two experiments; frequencies from one representative experiment quantified. *P = 0.0237, **P = 0.0028 (unpaired *t* test). **(D)** WT (*n* = 9) and B-*Fip200*^*−/−*^ (*n* = 6) mice were immunized with 30 μg NP_29_-KLH with Alhydrogel and sacrificed at day 42. Representative flow cytometry plots and frequency of B220^+^CD38^+^IgD^lo^NP^+^ memory B cells from one experiment. Left to right: ***P = 0.0002, ***P = 0.0004 (unpaired *t* test). **(E)** At day 42, WT (*n* = 4) and B cell–specific FIP200-deficient mice (*n* = 4) were boosted with 30 μg NP_29_-KLH in PBS. Anti-NP_29_– and anti-NP_7_–specific IgG ASCs in both spleen and BM at day 49 by ELISPOT. Plots show values for individual mice (symbols) and mean ± SD (bars). Top: *P = 0.0255; bottom: *P = 0.0272, **P = 0.0031 (unpaired *t* test). Performed twice, one replicate shown.

To evaluate the contribution of FIP200 to the humoral immune response, we immunized B-*Fip200*^−/−^ and WT mice with a T cell–dependent antigen, 4-hydroxy-3-nitrophenyl acetyl (NP) conjugated to keyhole limpet hemocyanin (KLH); mice were boosted at day 85. Serum collected over the course of the response indicated that both low-affinity (anti-NP_29_) and high-affinity (anti-NP_7_) IgG, while higher at day 11, decreased more rapidly in B-*Fip200*^−/−^ than WT mice over time. Furthermore, the titers produced after boosting were severely diminished as well, suggesting potential defects in both memory and plasma cell production (Fig. 1, A and B). Given the effects of FIP200 deficiency in B cells on antibody response, we further interrogated GC and plasma cell formation in response to NP_29_-KLH (Fig. 1 C, upper). The percentage of GC B cells (B220^+^Fas^+^CD38^−^) on day 11 after immunization was significantly reduced in the spleens of B-FIP200 KO mice relative to WT mice, as was the percentage of plasma B cells (B220^lo^IgD^−^CD138^+^) (Fig. 1 C, lower). Since the boosting immune response was weak in B-*Fip200*^−/−^ mice, we analyzed the antigen-specific memory B cells at day 42 and found an ∼80% reduction of antigen-specific memory B cells (B220^+^CD38^+^IgD^lo^NP^+^) in B-*Fip200*^−/−^ compared with WT mice (Fig. 1 D). We also assessed plasmablast/plasma cell differentiation by enzyme-linked immunospot (ELISPOT) and found that the numbers of both high (NP_7_-reactive)- and low (NP_29_-reactive)-affinity NP-specific IgG^+^ antibody-secreting cells (ASCs) were significantly depleted, relative to WT, in both spleens and BM of B-*Fip200*^−/−^ mice on day 49 after immunization (Fig. 1 E). Together, these results indicate that the humoral immune systems of B-*Fip200*^−/−^ mice are deficient in responding to both first-encounter and boost antigens, in line with prior work on memory and autophagy, and furthermore suggest that FIP200 regulates the response from ASCs.

### FIP200 is essential to plasma cell maintenance

**Figure 2.**
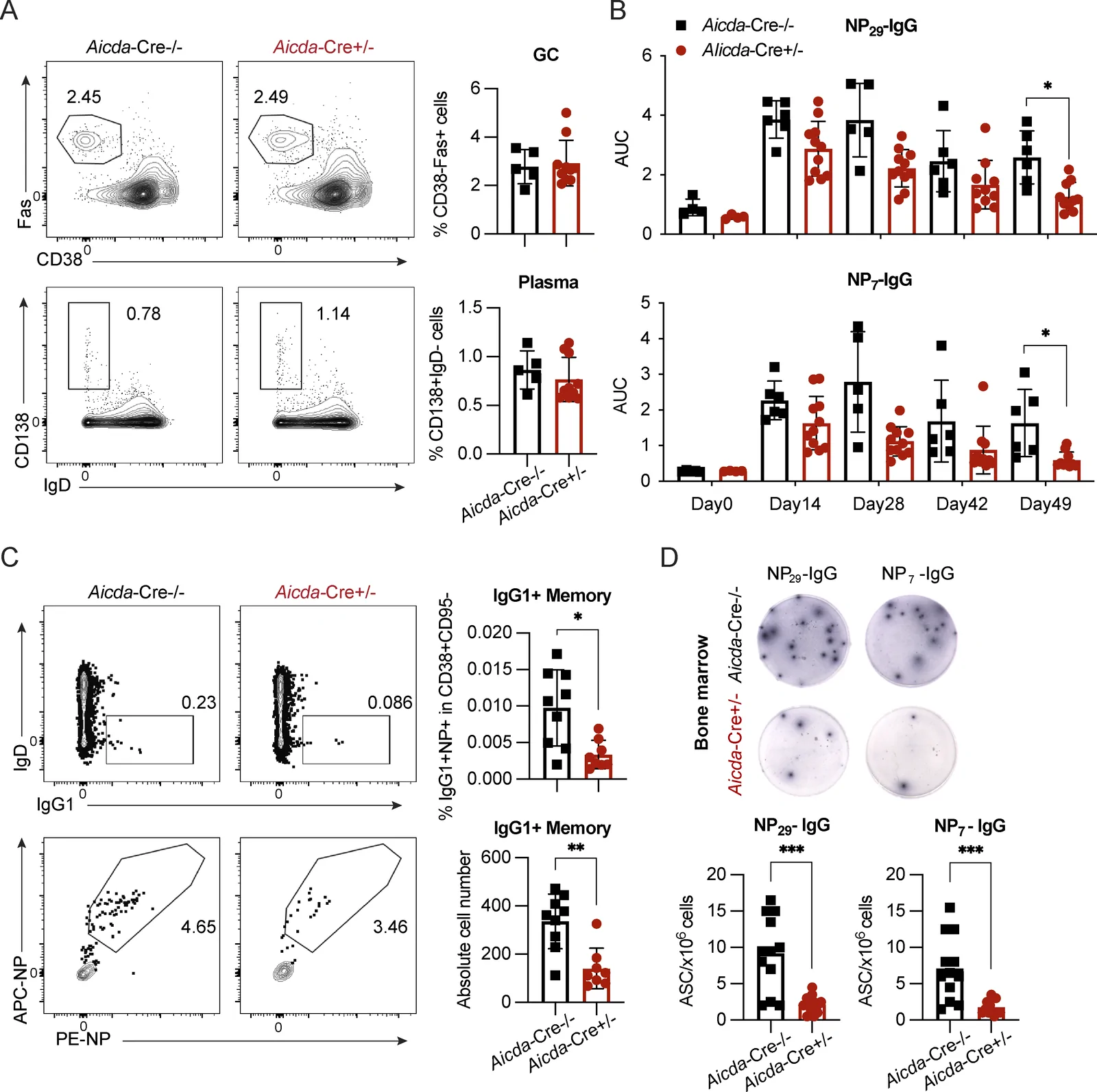
**FIP200 is required for plasma but not GC maintenance. (A)** 5 WT and 10 *Aicda-Cre*^+/−^ FIP200-deficient mice were immunized with 50 μg NP_29_-KLH with Imject Alum and sacrificed at day 11. Two replicates were performed; representative flow cytometry plots and frequency of Fas^+^CD38^−^ GC and CD138^+^ plasma B cells from one experiment shown. **(B)** WT and *Aicda-Cre*^+/−^ FIP200-deficient mice were immunized with 30 μg NP_29_-KLH with Imject Alum. Mouse serum was collected at regular intervals, and anti-NP_29_ (upper)– or anti-NP_7_ (lower)–specific IgG antibodies were detected by ELISA. Plots show values for individual mice (symbols) and mean ± SD (bars). Top: *P = 0.13; bottom: *P = 0.043. **(C)** WT (*n* = 9) and *Aicda*-*Cre*^+/−^ Fip200-deficient (*n* = 8) mice were immunized with 30 μg NP_29_-KLH with Alhydrogel and sacrificed at day 42. Representative flow cytometry plots and frequency of B220^+^CD38^+^IgD^lo^IgG1^+^NP^+^ memory B cells from one experiment. Top to bottom: *P = 0.0111, **P = 0.0025 (unpaired *t* test). **(D)** WT (*n* = 4) and *Aicda-Cre*^+/−^ FIP200-deficient (*n* = 4) mice were immunized with 30 μg NP_29_-KLH with Imject Alum and boosted with 30 μg NP_29_-KLH in PBS at day 42. Anti-NP_29_– and anti-NP_7_–specific IgG ASCs in BM at day 49 by ELISPOT. Plots show values for individual mice with triplicates. Significant (α = 0.05) P values were determined by an unpaired *t* test. Left to right: ***P = 0.0007, ***P = 0.0003.

To better understand the role FIP200 plays in activated B cells and their differentiation, we crossed *Fip200*^*flox/flox*^ mice with mice expressing *Aicda*-Cre. As activation-induced cytidine deaminase (AID) is predominantly expressed in B cells after activation, and is required for class switching and SHM (Muramatsu et al., 1999; Muramatsu et al., 2000; Roco et al., 2019), the *Aicda*-Cre cross leads to *Fip200* deletion early after activation; *Fip200*^*flox/flox*^*Aicda*-Cre^−/−^ (*Aicda*-Cre^−/−^) mice again served as the control for these experiments. In contrast to the B-*Fip200*^−/−^ mouse line, *Fip200*^*flox/flox*^*Aicda*-Cre^+/−^ (*Aicda-*Cre^*+/−*^) mice were, in terms of both GC and plasma B cells, indistinguishable from *Aicda*-Cre^−/−^ at 11 days after NP_29_-KLH immunization (Fig. 2 A), suggesting that maintaining *Fip200* expression until activation allowed normal GC formation and early plasma cell responses, in contrast to the full B cell KO. However, as with the full B cell KO, both low- and high-affinity class-switched IgG titers were attenuated in *Aicda-*Cre^*+/−*^, particularly at later time points (Fig. 2 B). Antigen-specific IgG1 memory B cells (B220^+^CD38^+^IgG1^+^NP^+^) were also diminished by ∼60% in *Aicda-*Cre^*+/−*^ mice relative to *Aicda-*Cre^−/−^ mice at day 42 (Fig. 2 C). Furthermore, compared with *Aicda*-Cre^−/−^ mice, the number of both low- and high-affinity antigen-specific IgG^+^ ASCs in the BM of *Aicda-*Cre^*+/−*^ mice was significantly lower on day 49 after immunization (Fig. 2 D). These results indicate that FIP200 is essential for humoral responses *in vivo* and for memory and long-term plasma cell maintenance.

### FIP200 regulates B cell proliferation, differentiation, and survival

**Figure 3.**
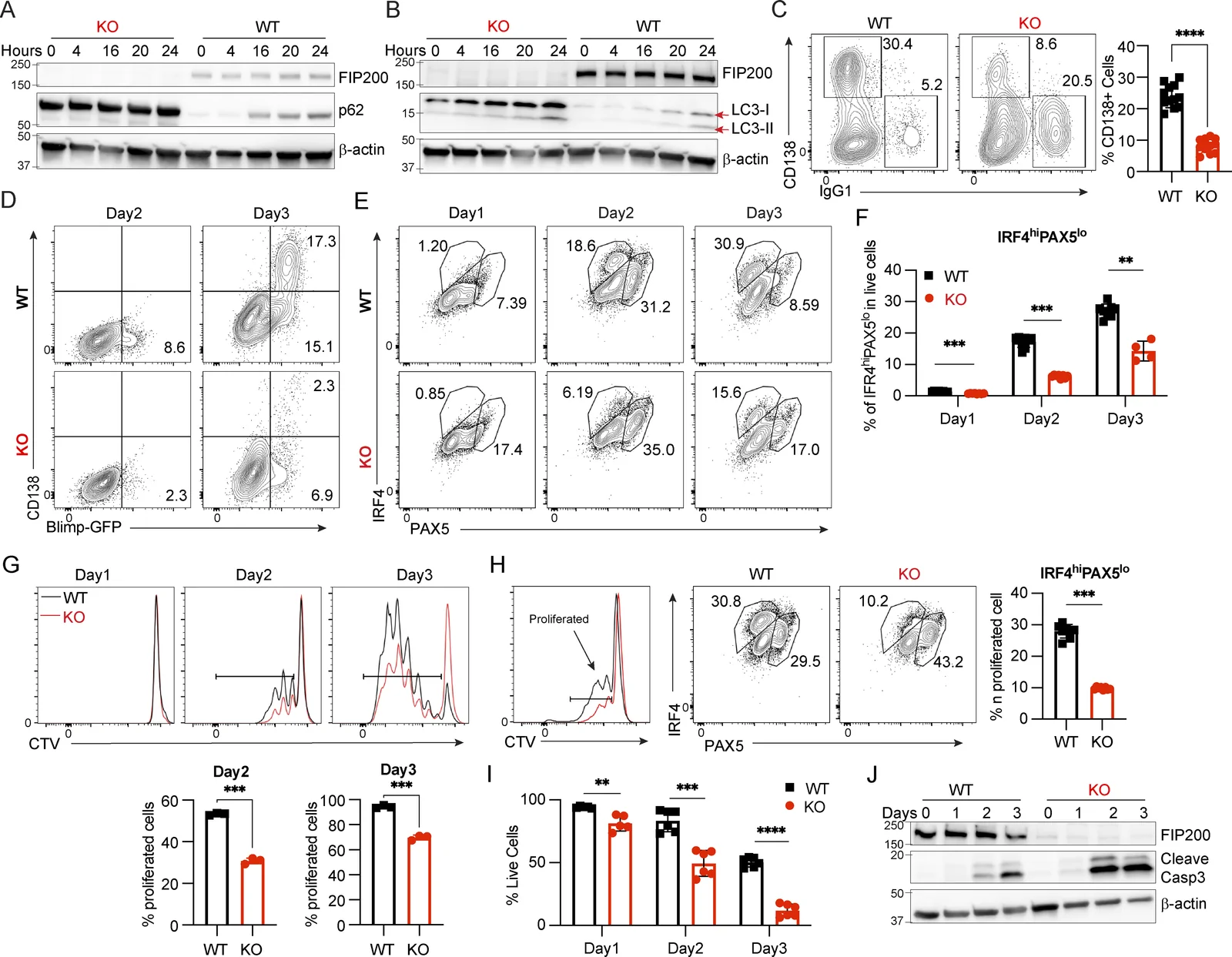
***Fip200*** ^***−/−***^ **B cells showed decreased proliferation, plasma differentiation, and survival *in vitro*.** B cells isolated from WT or B-*Fip200*^*−/−*^ mouse spleens were cultured with IL4 (10 ng/ml) + LPS (5 μg/ml). **(A and B)** Expression levels of p62 (A) and LC3 (B) were detected in WT and *Fip200*^*−/−*^ B cells upon stimulation at 0, 4, 16, 20, and 24 h. Representative data from one of at least two experimental replicates are shown. **(C)** Representative plots and the corresponding quantifications of plasma cells of WT and *Fip200*^*−/−*^ B cells after 3 days of culture. Data are combined from two independent experiments with at least three mice in each group, and samples are run in triplicate. Significant (α = 0.05) P values were determined by unpaired, two-tailed Student’s *t* test; ****P < 0.0001. **(D)** WT and *Fip200*^*−/−*^ B cells expressing Blimp-GFP were stimulated by IL4+LPS. Blimp-GFP^+^ (day 2) or Blimp-GFP^+^CD138^+^ (day 3) populations were checked by FACS. *N* = 5 biological replicates, with representative data shown from one mouse. **(E and F)** Representative plots (E) and the corresponding quantifications (F) of the IRF4^hi^PAX5^lo^ population of WT and *Fip200*^*−/−*^ B cells on days 1–3 of IL4 + LPS activation. Two independent experiments were performed. Representative data from one experiment with cells originating from four mice per group with two cultures generated per mouse are shown, except for KO, day 3, from which only one culture was obtained. Significant P values were determined by an unpaired *t* test. Left to right: ***P = 0.0002, ***P = 0.0002, **P = 0.0040. **(G)** Proliferation of WT and *Fip200*^*−/−*^ B cells upon stimulation by IL4+LPS was detected by FACS at days 1–3. Left to right: ***P = 0.0003, ***P = 0.0002. **(H)** Representative plots and the corresponding quantifications of IRF4^hi^PAX5^lo^ population in proliferated populations (CTV^lo^) of WT or *Fip200*^*−/−*^ B cells on day 2 of IL4 + LPS activation. Two independent experiments were performed. Representative data from one experiment with cells originating from four mice per group with two cultures generated per mouse are shown. Significant P values were determined by an unpaired *t* test. ***P = 0.0002. **(I)** WT and *Fip200*^*−/−*^ B cells were stimulated by IL4+LPS, and cell survival rate was detected by FACS on days 1–3. Left to right: **P = 0.0096, ***P = 0.0001, ****P < 0.0001 (unpaired *t* test). *N* = 3 biological replicates, with representative data shown from one mouse. **(J)** Cleaved caspase-3 was detected in WT and *Fip200*^*−/−*^ B cells after stimulation by IL4+LPS at days 2–3. *N* = 2 biological replicates, with representative data shown from one mouse. Source data are available for this figure: SourceData F3.

**Figure S2.**
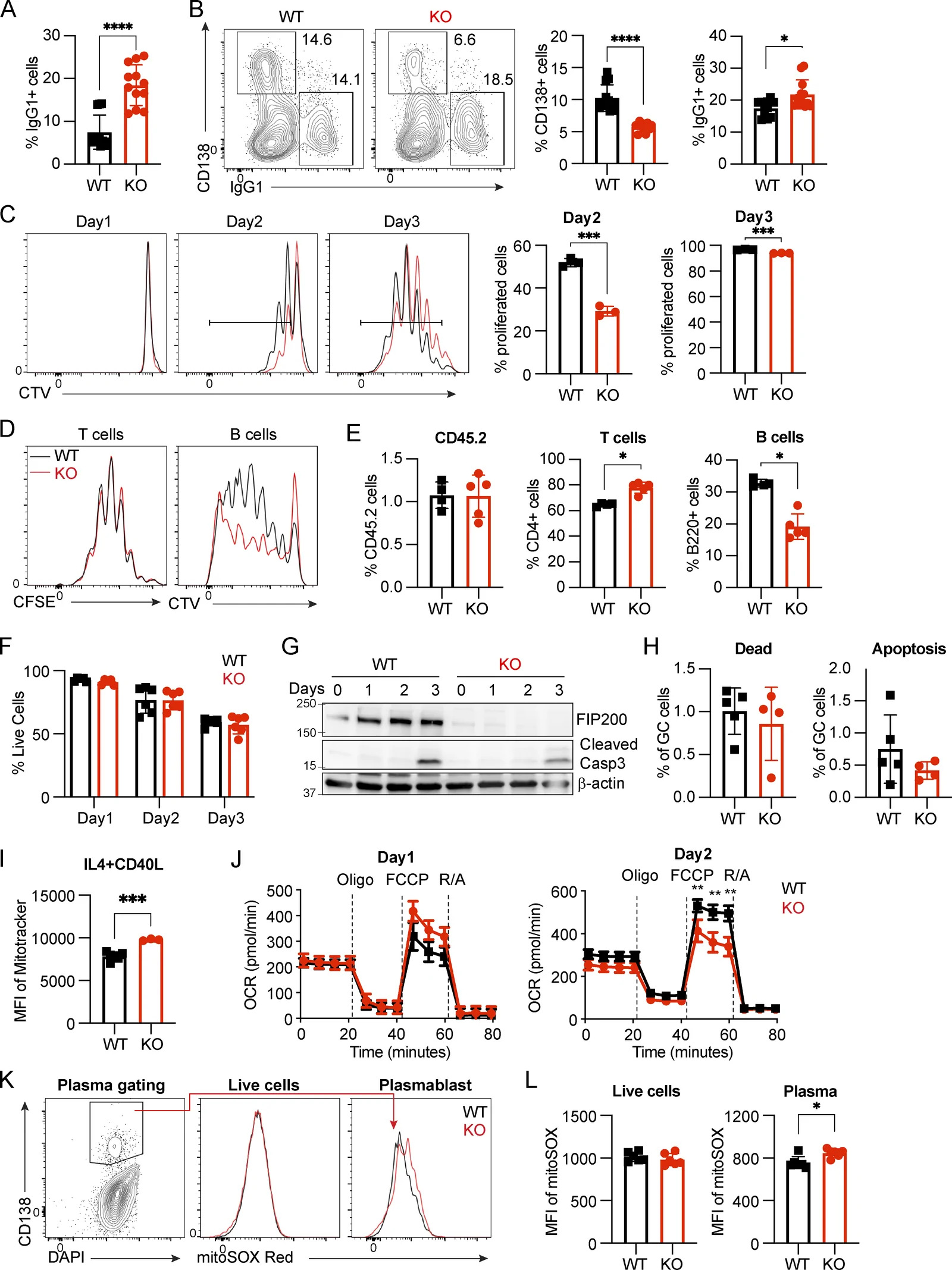
***Fip200*** ^***−/−***^ **B cells show decreased proliferation and plasma differentiation, and increased dysfunctional mitochondria mass upon IL4+CD40L stimulation, related to Figs. 3 and 4. (A)** Quantification of IgG1^+^ cells from WT and FIP200-deficient B cells cultured in the presence of IL4+LPS for 3 days. Data are combined from two independent experiments with four mice in each group, and samples are run in triplicate. Significant P values were determined by an unpaired *t* test. ****P < 0.0001. **(B)** Representative plots and the corresponding quantifications of plasma cells and IgG1^+^ of WT and FIP200-deficient B cells were cultured with IL4 (10 ng/ml) + CD40L (50 ng/ml) at day 3. Data are combined from two independent experiments with four mice in each group, and samples are run in triplicate. Significant P values were determined by an unpaired *t* test. ****P < 0.0001, *P = 0.0250. **(C)** Proliferation of WT (*n* = 1–3) and *Fip200*^*−/−*^ (*n* = 1–3) B cells upon stimulation of IL4+CD40L was detected by FACS at days 1–3. Three independent experiments were performed with representative data from one experiment with three mice per group shown. Left to right: ***P = 0.0002, ***P = 0.0008 (unpaired *t* test). **(D and E)** MD4 WT or *Fip200*^*−/−*^ B cells were stained with CTV, and OT-II T cells were stained with CFSE, then adoptively transferred to CD45.1^+^ recipient mice (*n* = 5). Mice were immunized at day 1 with OVA-HEL by intravenous injection. Proliferation of OT-II T and MD4 cells B cells was detected by FACS at day 4. Left to right: *P = 0.0159, *P = 0.0159 (unpaired *t* test). **(F)** Cell survival rate of WT (*n* = 1–3) and *Fip200*^*−/−*^ (*n* = 1–3) B cells upon stimulation of IL4+CD40L was detected by FACS at days 1–3. Three independent experiments were performed with representative data from one experiment with three mice per group shown. **(G)** Cleaved caspase-3 was detected in WT and *Fip200*^*−/−*^ B cells upon stimulation by IL4+CD40L at days 1–3. Two biological replicates were performed with data from one mouse shown. **(H)** WT (*n* = 5) and B-*Fip200*^*-/-*^ (*n* = 4) mice were immunized with 50 μg NP_29_-KLH with Imject Alum and sacrificed at day 12. Quantifications of dead (Live/Dead Blue^+^ Annexin V^+^) and apoptosis (Live/Dead Blue^−^ Annexin V^+^) population in GC B cells (B220^+^Fas^+^CD38^−^) by FACS. **(I)** Mitochondrial mass in day 3 IL4+CD40L-activated WT (*n* = 2–5) and *Fip200*^*−/−*^ (*n* = 3) B cells stained with MitoTracker Deep Red, ***P = 0.0001. Three independent experiments were performed with one shown. **(J)** OCR was measured by Seahorse XF analyzer (*n* = 4) for activated B cells at day 1 (left) and day 2 (right). FCCP is a mitochondrial uncoupling agent. Oligo., oligomycin; R/A, rotenone/antimycin. Data are representative of at least two independent experiments with at least three mice in each group. Left to right: **P = 0.0079, **P = 0.0030, **P = 0.0016 (unpaired *t* test). **(K and L)** WT and *Fip200*^*−/−*^ B cells were stimulated by IL4+CD40L and stained with MitoSOX Red and gated on CD138^+^ population. Representative plots (K) and the corresponding quantifications (L) of total live cells (left) or plasma cells (right) of WT and *Fip200*^*−/−*^ B cells. Data are combination of two independent experiments with more than two mice in each group. Significant P values were determined by an unpaired *t* test. *P = 0.0125. Source data are available for this figure: SourceData FS2.

To dissect the molecular mechanisms driving the detrimental effects of FIP200 knockout on plasma cell formation and maintenance, we examined autophagy *in vitro*. Splenic naïve B cells were isolated from B-*Fip200*^−/−^ or WT mice and activated by IL4+LPS. The autophagy receptor p62 and LC3-1 both accumulated in *Fip200*^*−/−*^ B cells at the naïve stage and after activation (Fig. 3, A and B), suggesting that the autophagy pathway was damaged in *Fip200*^*−/−*^ B cells, as also observed in WIPI2-KO mice (Martinez-Martin et al., 2017). Consistent with the *in vivo* data (Fig. 1 D), after 3 days of culturing with IL4+LPS, *Fip200*^*−/−*^ B cells showed a ∼60% reduction in differentiation into plasmablast populations (CD138^+^) (Fig. 3 C) and a ∼140% increase in the class-switched population (IgG1^+^) relative to WT B cells (Fig. S2 A). Similar reductions in plasma differentiation and increases in class switching were also observed after IL4+CD40L stimulation (Fig. S2 B).

To determine whether alterations in FIP200 affect the plasma transcriptional program, which is driven by B lymphocyte-induced maturation protein 1 (*Blimp-1/Prdm1*) (Shapiro-Shelef et al., 2003), we crossed B-*Fip200*^−/−^ mice with Blimp-GFP mice (Kallies et al., 2004). After culturing the B-*Fip200*^−/−^-Blimp-GFP B cells, we measured BLIMP-GFP expression and found *Fip200-*deficient B cells showed significant reductions in BLIMP-GFP–positive cells at day 2 (Fig. 3 D). We then examined another plasma differentiation transcription factor, IRF4, which targets the *Prdm1* locus and activates its transcription (Nutt et al., 2011; Ochiai et al., 2013). Using intracellular staining, we observed upon activation an increase in IRF4 expression from day 1 to day 3 in both WT and FIP200 KO B cells. PAX5 expression also increased in both groups from day 1 to day 2, and then decreased on day 3. However, the preplasma, IFR4^hi^PAX5^lo^ population among FIP200 KO B cells at day 2 showed a ∼66% reduction compared with WT B cells (Fig. 3, E and F).

We examined proliferation *in vitro* upon stimulation using CellTrace Violet (CTV) dye labeling and found it significantly impaired in the knockout after IL4+LPS (Fig. 3 G) or IL4+CD40L (Fig. S2 C) stimulation, potentially due to the inhibition of mTOR signaling (Gan et al., 2005). To validate this observation *in vivo*, we adoptively transferred CD45.2^+^CFSE^+^ OT-II T cells with CD45.2^+^CTV^+^ FIP200 KO or WT MD4 B cells into CD45.1 recipient mice and immunized recipients with the OVA-HEL complex intravenously on day 1. At day 4, we found similar proliferation of T cells in both groups, but diminished proliferation of FIP200 KO MD4 B cells compared with WT (Fig. S2 D). We observed ∼40% fewer FIP200 KO B cells than WT B cells and a slight increase of T cell populations in the FIP200 KO B group (Fig. S2 E).

As both proliferation and differentiation appeared to be defective in knockout cells, we examined the capacity of proliferated B cells to differentiate *in vitro*. Upon IL4+LPS stimulation at day 2, proliferated (CTV^lo^ population) FIP200 KO B cells showed a ∼66% reduction of the IFR4^hi^PAX5^lo^ population compared with that in WT B cells (Fig. 3 H), demonstrating that both proliferation and plasma differentiation likely contributed to diminished *Fip200*^*−/−*^ B cell plasma populations.

We then examined cell death after stimulation. After 3 days of IL4+LPS stimulation, only approximately one fourth as many *Fip200*^*−/−*^ B cells as WT B cells survived (Fig. 3 I), though no similar gap arose after IL4+CD40L activation (Fig. S2 F). To determine the cause of this massive cell death in FIP200 KO B cells after IL4+LPS stimulation, we interrogated apoptotic signals in the autophagy-deficient B cells and found a dramatic increase in the levels of cleaved caspase-3 at days 2 and 3 (Fig. 3 J). The lack of an equivalent increase of cell death (Fig. S2 F) and cleavage of caspase-3 after IL4+CD40L activation (Fig. S2 G) suggested a unique role of autophagy in B cell survival after activation through the TLR4 signaling pathway. To test this hypothesis *in vivo*, B-*Fip200*^−/−^ and WT mice were immunized with a T cell–dependent antigen, NP_29_-KLH, and stained with Annexin V. In line with the *in vitro* IL4+CD40L experiments, few dead or apoptotic cells were observed among GC B cells at day 12 and the populations did not differ between *Fip200*^*−/−*^ and WT mice (Fig. S2 H). Thus, we determined that FIP200 plays an important role in autophagy in B cells, regulating proliferation and plasma cell differentiation, but is only critical for B cell survival after TLR4 stimulation.

### FIP200-deficient B cells are prone to accumulate dysfunctional mitochondria

**Figure 4.**
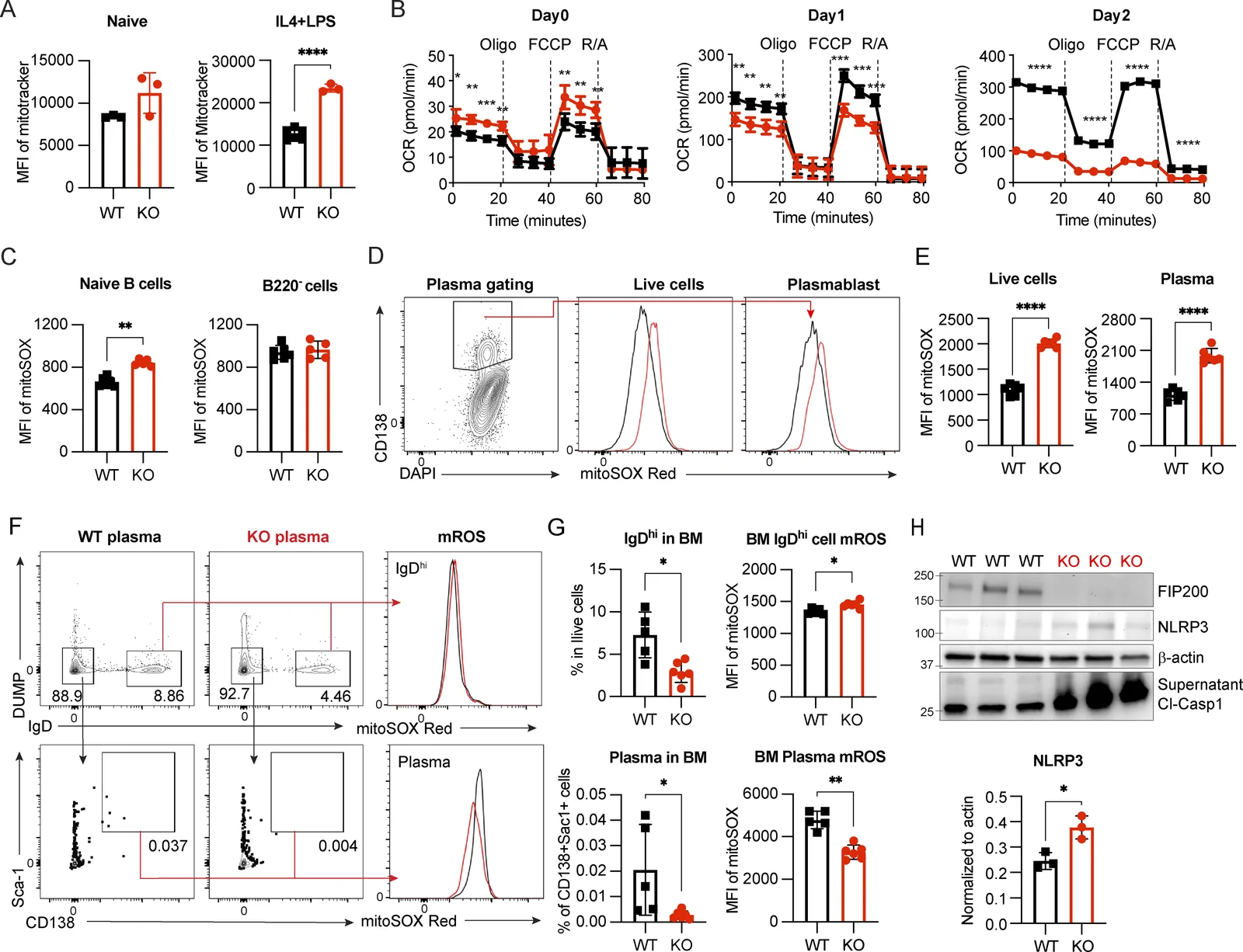
**Dysfunctional mitochondria accumulate in *Fip200*** ^***−/−***^ **B cells. (A)** Mitochondrial mass in naïve (left, WT, *n* = 3; and *Fip200*^*−/−*^, *n* = 3) and day 3 IL4+LPS-activated (right, WT, *n* = 6; and *Fip200*^*−/−*^, *n* = 3) B cells stained with MitoTracker Deep Red, ****P < 0.0001 (unpaired *t* test). Representative data of at least two experimental replicates shown. **(B)** OCR measured by Seahorse XF analyzer for activated B cells (four samples per group) at day 0 (left), day 1 (middle), and day 2 (right). FCCP is a mitochondrial uncoupling agent. Oligo., oligomycin; R/A, rotenone/antimycin. From left to right: day 0, *P = 0.0325, **P = 0.0014, ***P = 0.0007,**P = 0.0022, **P = 0.0083, **P = 0.0026, **P = 0.0057; day 1, **P = 0.0027, **P = 0.0041, **P = 0.0051, **P = 0.0058, ***P = 0.0002, ***P = 0.0003, ***P = 0.0004; day 2, ****P < 0.0001, ****P < 0.0001, ****P < 0.0001, ****P < 0.0001 (unpaired *t* test). **(C)** Splenocytes from WT (*n* = 6) and B-*Fip200*^*−/−*^ (*n* = 5) mice were stained with MitoSOX Red and naïve splenic B cells (left) and B220^−^ cells (right) detected by FACS, **P = 0.0022 (unpaired *t* test). **(D and E)** WT and *Fip200*^*−/−*^ B cells were stimulated by IL4+LPS and stained with MitoSOX Red and gated on CD138^+^ population. Representative plots (D) and the corresponding quantifications (E) of total live cells (left) or plasma cells (right) of WT and *Fip200*^*−/−*^ B cells. Data are representative of at least two independent experiments run with two mice per group. Representative data from one experiment are shown. Left to right: ****P < 0.0001, ****P < 0.0001 (unpaired *t* test). **(F and G)** BM cells from WT (*n* = 5) and B-*Fip200*^*−/−*^ (*n* = 6) mice were stained with MitoSOX and gated on IgD^hi^ cells (DUMP^−^IgD^hi^) and plasma cells (DUMP^−^IgD^−^Sca-1^+^CD138^+^). Representative plots (F) and the corresponding quantifications (G) of IgD^hi^ cells (top left), mROS of IgD^hi^ cells (top right), plasma cells (bottom left), or mROS of plasma (bottom right) of WT and *Fip200*^*−/−*^ B cells. Two independent experiments were performed; representative data from one experiment are shown. Top left to right: *P = 0.0173, *P = 0.0173; bottom left to right: *P = 0.0173, **P = 0.0043 (unpaired *t* test). **(H)** Expression level of NLRP3 in WT and *Fip200*^*−/−*^ B cells and cleaved caspase-1 in the supernatant upon IL4+LPS stimulation at day 2. This experiment was performed twice, with results from one independent run shown; each lane represents one mouse. Significant (α = 0.05) P values were determined by an unpaired *t* test. *P = 0.0182. Source data are available for this figure: SourceData F4.

Mitochondrial status is a key determinant of plasma cell differentiation (Jang et al., 2015; Sandoval et al., 2018), and FIP200-involved macroautophagy is one way to remove dysfunctional mitochondria. We therefore investigated mitochondrial homeostasis in *Fip200*^*−/−*^ B cells. We found increased mitochondrial mass in naïve and activated *Fip200*^*−/−*^ B cells 3 days after stimulation by IL4+LPS (Fig. 4 A) or IL4+CD40L (Fig. S2 I). To determine the functionality of this accumulated mitochondrial mass, we examined mitochondrial respiration capacity by Seahorse. Naïve *Fip200*^*−/−*^ B cells showed slightly higher oxygen consumption rate (OCR) compared with WT B cells, consistent with their increased mitochondrial mass (Fig. 4 B). Upon activation by IL4+LPS, maximum OCR was lower than that in WT B cells at day 1 and decreased even further at day 2 (Fig. 4 B). After IL4+CD40L stimulation, OCR of WT and *Fip200*^*−/−*^ on day 1 was similar, but *Fip200*^*−/−*^ OCR was again significantly diminished relative to WT on day 2 (Fig. S2 J). We also used MitoSOX Red to stain mROS in both WT and *Fip200*^*−/−*^ B cells. Naïve B cells (B220^+^IgD^hi^) from the spleens of B-*Fip200*^−/−^ mice had higher mROS than naïve B cells from WT mice, while mROS in B220^-^ cells was similar in B-*Fip200*^−/−^ and WT mice (Fig. 4 C). After 3-day culturing of isolated B cells, mROS levels were higher in both the live and plasma populations (CD138^+^) of FIP200 KO B cells than in WT after IL4+LPS stimulation (Fig. 4, D and E); after IL4+CD40L activation, in contrast, while mROS in *Fip200*^*−/−*^ plasma cells increased somewhat relative to WT, it was indistinguishable in the total live-cell populations (Fig. S2, K and L). In the BM, IgD^hi^ mature B cells were diminished in B-*Fip200*^−/−^ mice relative to WT, and the mROS levels in BM IgD^hi^ mature B cells in B-*Fip200*^−/−^ mice were slightly higher than in WT (Fig. 4, F and G). B-*Fip200*^−/−^ mice had far fewer plasma cells compared with WT mice; BM plasma cells from B-*Fip200*^−/−^ mice had significantly lower mROS than those from WT (Fig. 4, F and G). Thus, *Fip200* ablation was associated with accumulation of dysfunctional mitochondrial mass and resulted in increased mROS in naïve and activated B cells, which lead to cell death in long term; the apparently contradictory decrease in mROS in the surviving BM plasma cells may suggest that FIP200 KO B cells could not sustainably accommodate increased mROS, and higher mROS cells did not survive to be counted in the BM.

TLR4 signaling, followed by increased mROS, induces the NLRP3 inflammasome, which leads to the release of proinflammatory cytokines and pyroptotic cell death (Swanson et al., 2019). Autophagy proteins are critical to inhibit NLRP3 activation (Nakahira et al., 2011). Since significant cell death occurred in *Fip200*^*−/−*^ B cells activated by IL4+LPS, we investigated NLRP3 activation in B cells stimulated through the TLR4 signaling pathway. In *Fip200*^*−/−*^ B cells activated by IL4+LPS, NLRP3 accumulated and more cleaved caspase-1 was detected in the supernatant on day 2 (Fig. 4 H); this is consistent with previous results (Fig. 3 I) and suggests pyroptosis in *Fip200*^*−/−*^ B cells upon TLR4 activation. Taken together, these data indicated an accumulation of dysfunctional mitochondria and mROS in *Fip200*^*−/−*^ B cells upon activation. Furthermore, LPS stimulation induced NLRP3 inflammasome formation in B cells and autophagy proteins, such as FIP200, were vital for the clearance of the NLRP3 inflammasome and thus for B cell survival.

### FIP200 regulates mitophagy in activated B cells

**Figure 5.**
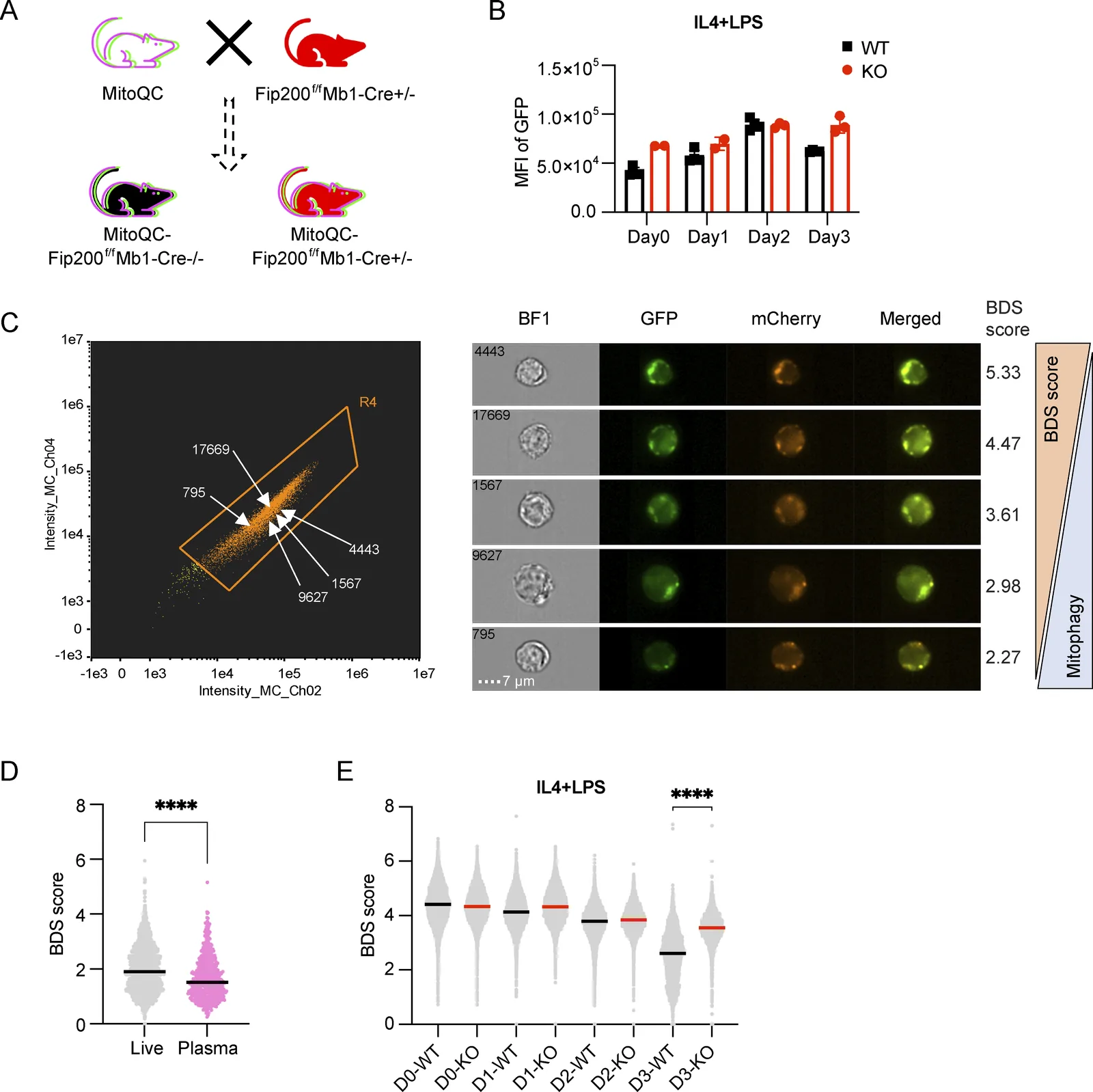
**FIP200 regulates mitophagy in B cells upon activation. (A)** Schematic breeding strategy to generate MitoQC-B-*Fip200*^*−/−*^ mice and their littermate controls. **(B)** Dynamic of the MitoQC-GFP expression level in WT and MitoQC-B-*Fip200*^*−/−*^ B cells on day 3 of IL4+LPS stimulation. Data are representative of at least three independent experiments (*n* = 2–3 mice per treatment). One representative experiment is shown. **(C)** Representative cells from one WT mouse to demonstrate BDS scoring approach in gate R4 (GFP^+^mCherry^+^). **(D)** Representative plots of BDS scores of total live cells and CD138^+^ plasma cells measured by ImageStream; data were analyzed by the Kolmogorov–Smirnov test, ****P < 0.0001. Cells were cultured independently from two WT mice, with summary data from one mouse-derived culture shown. **(E)** Representative plots of BDS scores of WT and MitoQC-B-*Fip200*^*−/−*^ B cells on day 3 of IL4+LPS stimulation; data were analyzed by the Kolmogorov–Smirnov test, ****P < 0.0001. The experiment was performed at least three times (*n* = 1–3); representative data from cultures derived from one WT and one knockout mouse are shown here.

**Figure S3.**
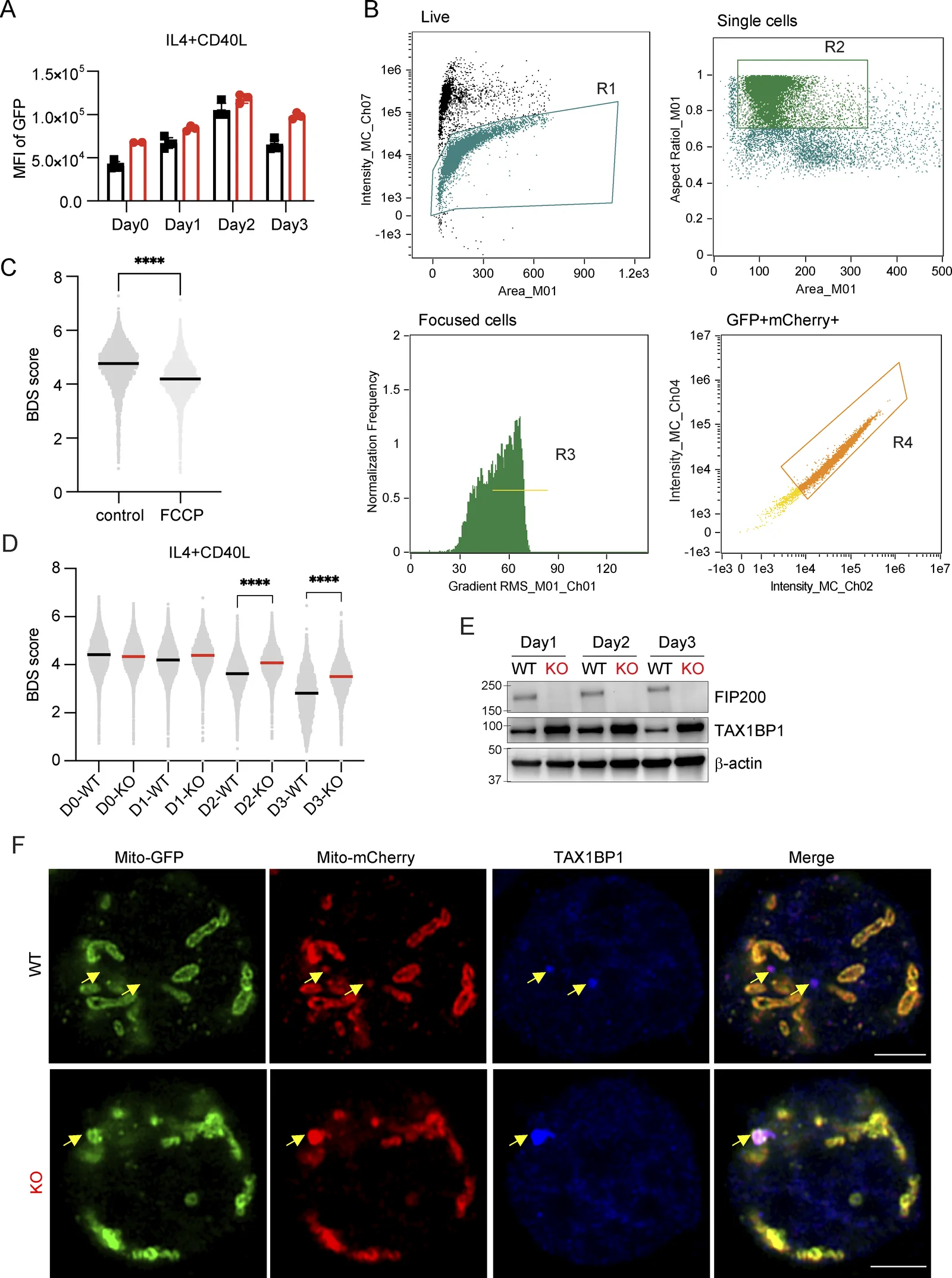
**FIP200 mediates mitophagy of TAX1BP1 binding mitochondria in B cells upon CD40L stimulation, related to Fig. 5. (A)** Dynamic of the MitoQC-GFP expression level in WT and MitoQC-B-*Fip200*^*−/−*^ B cells upon IL4+CD40L stimulation for 3 days. Data are representative of at least two independent experiments with three mice in each group. **(B)** Gating strategy of Live/single/focused/GFP^+^mCherry^+^ cells in ImageStream. **(C)** Representative plots of BDS score of MitoQC-B cells treated with FCCP or control (DMSO) for 30 min. Data are representative of at least two independent experiments; data from naïve B cells from one mouse per treatment are shown. **(D)** Representative plots of BDS score of WT and MitoQC-B-*Fip200*^*−/−*^ B cells upon IL4+CD40L stimulation for 3 days; data were analyzed by the Kolmogorov–Smirnov test, ****P < 0.0001. The experiment was performed independently three times, with one representative experiment shown. *n* = 2–3. **(E)** Expression of TAX1BP1 was detected in WT and *Fip200*^*−/−*^ B cells upon IL4+CD40L stimulation for 3 days. Two biological replicates were performed for WT and *Fip200*^*−/−*^; results from one shown. **(F)** Representative immunofluorescence Airyscan images with 90x magnification (Plan-Apochromat 50×/1.2 W objective, 1.8× magnification changer) of WT (*n* = 2; one shown) and MitoQC-B-*Fip200*^*−/−*^ (*n* = 2; one shown) B cells activated by IL4+CD40L at day 2 and stained with anti-TAX1BP1 antibody (blue), MitoQC-GFP (green), and MitoQC-mCherry (red). Scale bar, 3 μm. Yellow arrows point to TAX1BP1 aggregation in B cells. Source data are available for this figure: SourceData FS3.

Mitophagy is a subtype of autophagy for the removal of damaged mitochondria (Palikaras et al., 2018). FIP200 not only participates in canonical autophagy but also interacts with NDP52 to mediate mitophagy in mammalian cells (Dikic and Elazar, 2018; Vargas et al., 2019). To test whether the accumulation of dysfunctional mitochondria in *Fip200*^*−/−*^ B cells was the result of impeded mitophagy, we crossed MitoQC mice with *B-Fip200*^−/−^ mice and isolated splenic B cells (Fig. 5 A). These B cells expressed fused mCherry-GFP-FIS1^101-152^ protein, which is anchored on the outer mitochondrial membrane. When damaged mitochondria fuse with lysosomes, the low pH quenches GFP, leaving only the mCherry signal; reduced GFP and mCherry colocalization thus indicates mitophagy (McWilliams et al., 2016). First, we tracked the mean fluorescence of GFP and found MitoQC-B-*Fip200*^−/−^ cells accumulated GFP fluorescence signal in both naïve and at day 3 (Fig. 5 B and Fig. S3 A), indicating increased mitochondrial mass, consistent with previous data (Fig. 4 A and Fig. S2 I). Then, we used ImageStream to acquire the fluorescence of GFP and mCherry in activated WT B cells and determined the Bright Detail Similarity (BDS) Score of GFP and mCherry signals; BDS is higher when the GFP and mCherry signals are colocalized (Fig. 5 C and Fig. S3 B). To validate our system, we treated B cells with FCCP, which is known to induce mitophagy (Strappazzon et al., 2015). Within 30 min of treatment, the median BDS score decreased from ∼4.8 to ∼4.2 (Fig. S3 C), indicating active mitophagy. Previously, it was observed that activated B cells that differentiate into plasmablasts tend to have lower mitochondrial mass than those that undergo class switching (Jang et al., 2015). By analyzing the BDS in WT B cell scores at day 3 of IL4+LPS stimulation, we confirmed that the plasma (CD138^+^) population exhibited low BDS scores (∼1.5) compared with the live-cell (CD138^−^) population (∼1.9), indicating a relative increase in mitophagy in the plasmablast population (Fig. 5 D). We then tracked BDS score dynamics over time in WT and FIP200-deficient B cells after IL4+LPS or IL4+CD40L stimulation: mitophagy increased in frequency in both groups at day 3; however, in *Fip200*^*−/−*^ B cells, mitophagy was significantly impaired at this time point relative to WT (Fig. 5 E and Fig. S3 D).

Recent studies have indicated that FIP200 can promote degradation of aggregates through a pathway bypassing LC3-dependent lysosomal targeting (Huyghe et al., 2022; Ohnstad et al., 2020; Vargas et al., 2019). We found that TAX1BP1, a component of this pathway that binds to ubiquitin and FIP200 through its ubiquitin-binding zinc finger and SKICH domains, respectively (Ohnstad et al., 2020), accumulated in *Fip200*^*−/−*^ B cells after CD40L stimulation (Fig. S3 E). To determine whether TAX1BP1 plays a role in mitophagy, we performed intracellular staining of TAX1BP1 in WT and *Fip200*^*−/−*^ B cells at day 2 upon IL4+CD40L stimulation, and found that in WT B cells, most of the TAX1BP1 aggregates colocalized with the mCherry^+^GFP^−^ spots, suggesting that TAX1BP1 participated in mitophagy (Fig. S3 F, top). However, in *Fip200*^*−/−*^ B cells, TAX1BP1 accumulated on the mitochondria expressing both GFP and mCherry—indicating that this complex failed to fuse with the lysosome; the size and intensity of the aggregates were also greater in the KO cells than in the WT cells (Fig. S3 F, low). In sum, mitophagy, which occurred frequently in plasmablast cells, is tightly regulated by FIP200 through both LC3 lipidation-dependent and LC3 lipidation–independent pathways.

### FIP200 mediates plasma differentiation by regulating reactive oxygen species (ROS) and heme metabolism

**Figure S4.**
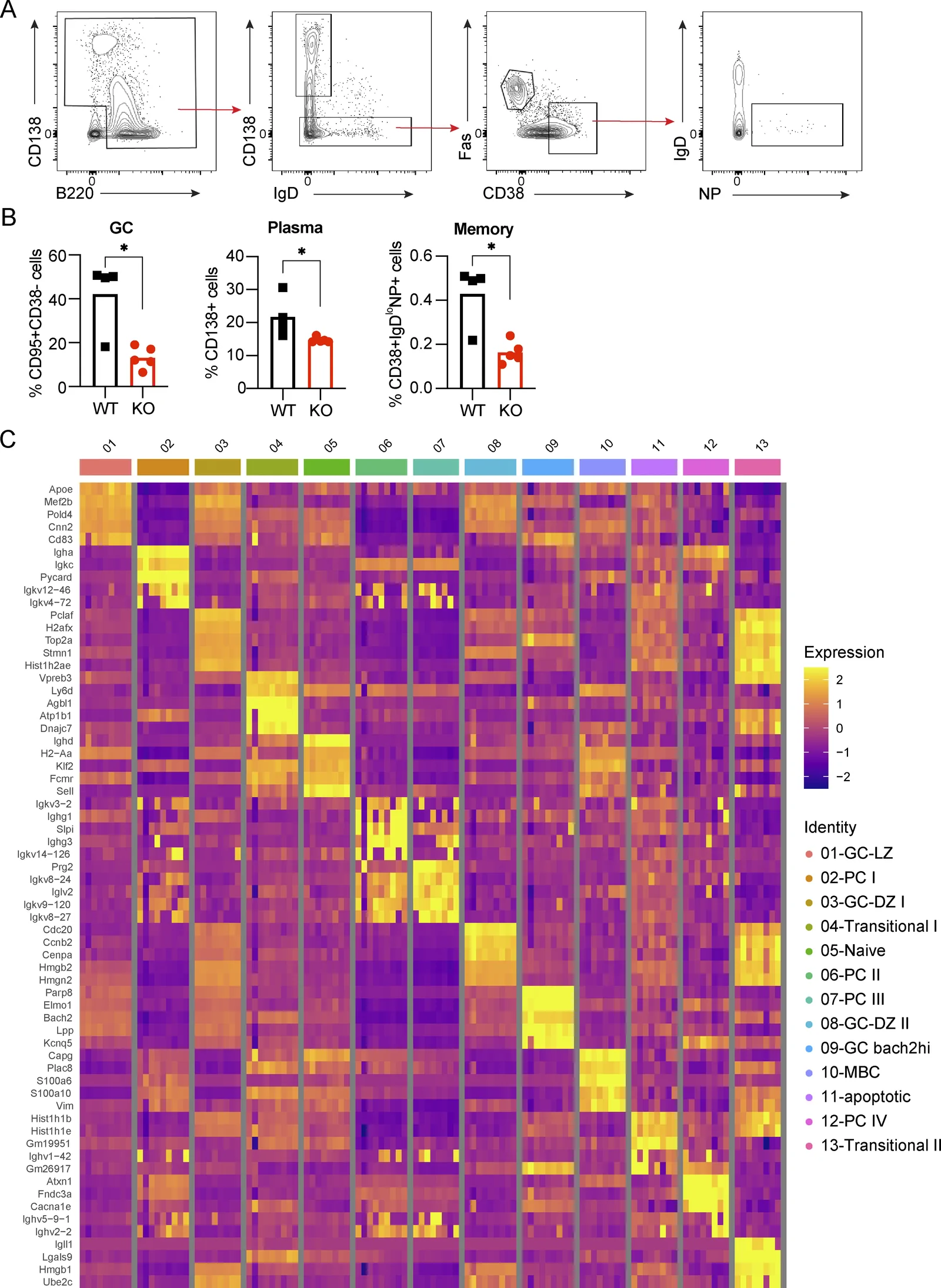
**Sample preparation for 10x single-cell RNA-Seq and cluster definition of B cells from WT and B-*Fip200*** ^***−/−***^ **mice, related to Fig. 6. (A)** Gating strategy of FACSorted plasma, GC, and memory B cell populations in IgD^+^ cell–depleted splenocytes from WT and B-*Fip200*^*−/−*^ mice. Naïve B cells (B220^+^IgD^hi^) were sorted from intact splenocytes. **(B)** Statistical analysis of GC, plasma, and memory B cell populations in IgD^+^ cell–depleted splenocytes from WT and B-*Fip200*^*−/−*^ mice, as in Fig. 6 A. Significant P values were determined by an unpaired *t* test. Left to right: *P = 0.0316, *P = 0.0405, *P = 0.0268. **(C)** Heat map of top five expressed genes in 13 clusters in Fig. 6 A.

**Figure 6.**
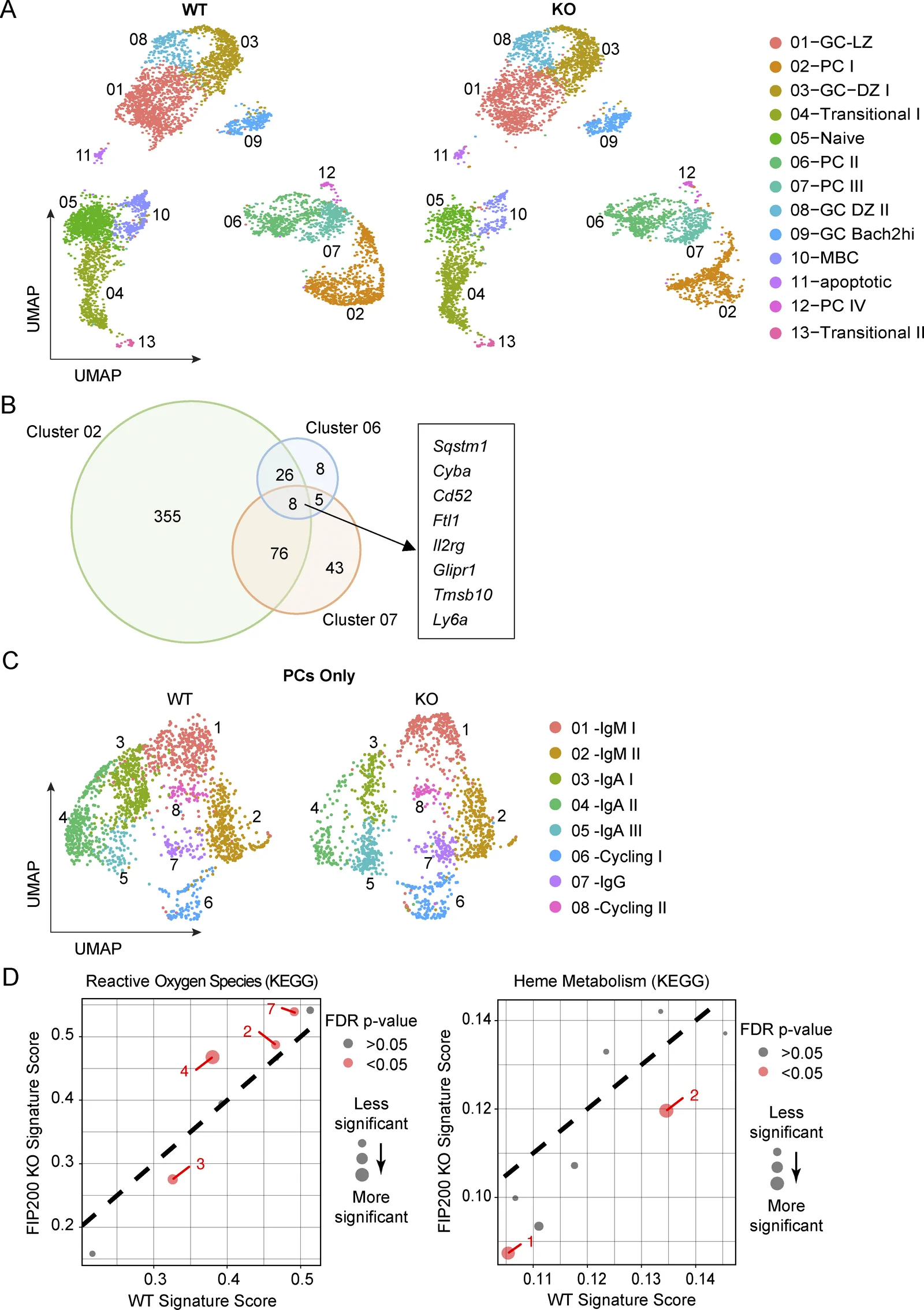
**FIP200 promotes plasma differentiation by balancing ROS and heme metabolism. (A)** Naïve, GC, memory, and plasma B cells FACSorted from WT and B-*Fip200*^*−/−*^ mice immunized with 50 μg NP_29_-KLH with Imject Alum at day 11. UMAP showing 13 B cell clusters and phenotypic identities in WT (left) and *Fip200*^*−/−*^ (right) groups. Representative of one experiment, *n* = 5 pooled B-*Fip200*^*−/−*^ mice and *n* = 4 pooled WT mice. **(B)** Venn diagram shows common genes upregulated in *Fip200*^*−/−*^ cells in clusters 2, 6, and 7 (major plasma populations), and genes upregulated in all three clusters compared with their WT control. **(C)** Subset and reclustering of B cells from plasma clusters 2, 6, 7, and 12 in A, colored by cluster. **(D)** Scatterplots showing average signature score, calculated in VISION, for curated KEGG pathways on a cluster-by-cluster basis in *Fip200*^*−/−*^ versus WT plasma cells for ROS (left) and heme metabolism (right). FDR, false discovery rate; UMAP, Uniform Manifold Approximation and Projection.

**Figure S5.**
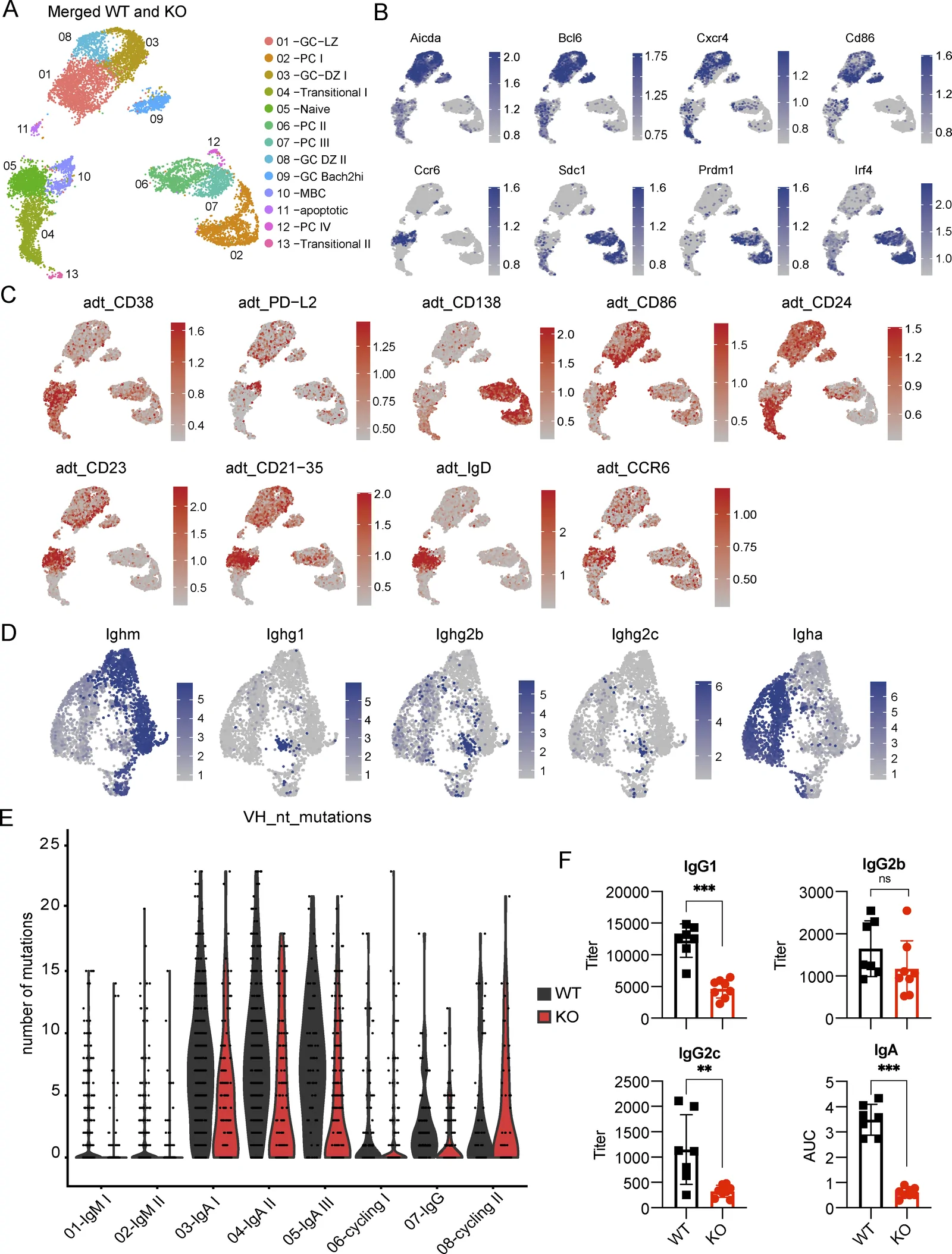
**Single-cell RNA-Seq of different B cell populations from WT and B-*Fip200*** ^***−/−***^ **mice, related to Fig. 6. (A)** UMAP of 13 B cell clusters and phenotypic identities in merged WT and *Fip200*^*−/−*^ groups in Fig. 6 A. **(B and C)** Feature plot of the B cells in A showing relative expression of signature genes labeled on top (B) and antibody-derived tag (ADT) antibody expression (C). Color scale represents centered natural log transformation across cells. **(D)** Distribution of immunoglobulin subclass mapped in the plasma population (Fig. 6 C) UMAP representation. The color scale represents centered natural log transformation across cells. **(E)** Plot of nucleotide mutation counts in the heavy chain variable region of different plasma clusters. Feature plot of cells for their CITE-Seq/ADT antibody expression. **(F)** NP-specific IgG1, IgG2b, IgG2c titers and IgA antibody level were detected by ELISA at day 56 in WT (*n* = 7) or B-*Fip200*^*−/−*^ (*n* = 8) mice after 30 μg NP_29_-KLH with Imject Alum immunization. Plots show values for individual mice (symbols) and means (bars). Significant P values were determined by an unpaired *t* test. Top: ***P = 0.0003; bottom: **P = 0.0093, ***P = 0.0003.

Having interrogated known associates of FIP200, we sought to broaden our understanding of its role in plasma differentiation by investigating transcription globally. First, splenocytes isolated from WT and B-*Fip200*^−/−^ mice 11 days after immunization with NP_29_-KLH were enriched for GC, plasma, and memory B cells by depleting non-B cells and IgD^hi^ B cells using magnetic beads. Those enriched samples were then barcoded and sorted for naïve, GC, plasma, and memory B cells, which were then mixed for single-cell RNA sequencing (RNA-Seq) (Fig. S4, A and B). We performed gene expression analysis using Seurat (Butler et al., 2018) and found 13 distinct clusters (Fig. 6 A and Fig. S4 C). The GC clusters (1, 3, 8, and 9) showed high expression of *Aicda* and *Bcl6*; *Cxcr4* and *Cd86*, which mark DZ and LZ B cells, respectively, displayed an inverted expression pattern (Fig. S5, A and B). The plasma clusters (2, 6, 7, and 12) showed high expression of *Sdc1*, *Prdm1*, and *Irf4*, which matched the antibody-derived tag (ADT) CD138 staining (Fig. S5, A–C). The naïve B cell clusters (4, 5, and 13) displayed positive hashtag staining of CD38, CD24, CD23, CD21/35, and IgD (Fig. S5, A and C). Fewer PD-L2^+^CCR6^hi^ memory B cells were sorted from B-*Fip200*^−/−^ mice compared with WT mice (Fig. 6 A, Fig. S4 B, and Fig. S5, A–C).

While few genes were differentially expressed between B-*Fip200*^−/−^ and WT GC and naïve B cell clusters, 480 differentially expressed genes were detected in plasma cell cluster 2, 58 were detected in plasma cell cluster 6, and 144 were detected in plasma cell cluster 7. Of those, 465 genes in cluster 2, 47 genes in cluster 6, and 132 genes in cluster 7 were upregulated in *Fip200*^*−/−*^ B cells compared with WT B cells. We then analyzed the gene set differentially expressed in all three clusters and identified eight genes consistently upregulated in B-*Fip200*^−/−^ plasma populations (Fig. 6 B). These commonly upregulated genes included two previously associated with autophagy and ROS pathways, *Sqstm1* and *Cyba*.

Within the plasma population, we identified eight subclusters by gene expression signatures (Fig. 6 C). We observed an unusual IgA population (clusters 2, 3, and 4) in both WT and KO; based on the number of mutated nucleotides in their heavy chain V regions relative to the IgG cluster (Fig. S5, D and E), this population may be a by-product of the rearing environment. The majority of IgG plasma in WT mice expressed IgG1, while in B-*Fip200*^−/−^ mice, the primary subclass expressed was IgG2b, in line with the day 56 NP-specific antibody titers (Fig. 6 C and Fig. S5, D and F). NP-specific IgG1 titers were significantly reduced in B-*Fip200*^−/−^ mice, while NP-specific IgG2b was similar between WT and B-*Fip200*^−/−^ mice (Fig. S5 F). To better understand the transcriptional differences between WT and B-*Fip200*^−/−^ plasma populations, we used VISION to analyze KEGG metabolic pathways and found ROS was upregulated in three of the B-*Fip200*^−/−^ plasma cell clusters (2, 4, and 7), while heme metabolism was downregulated in IgM^+^ B-*Fip200*^−/−^ plasma cell clusters (1 and 2) (Fig. 6 D). In sum, single-cell profiling confirmed that upon immunization by a T cell–dependent antigen, B-*Fip200*^−/−^ B cells differentiated less frequently into plasma and memory B cells and furthermore demonstrated that plasma cells specifically expressed distinctive IgG subclasses, displayed increased mROS, and had downregulated heme metabolism pathways.

### Hemin rescues plasma differentiation in *Fip200*^−/−^ B cells

**Figure 7.**
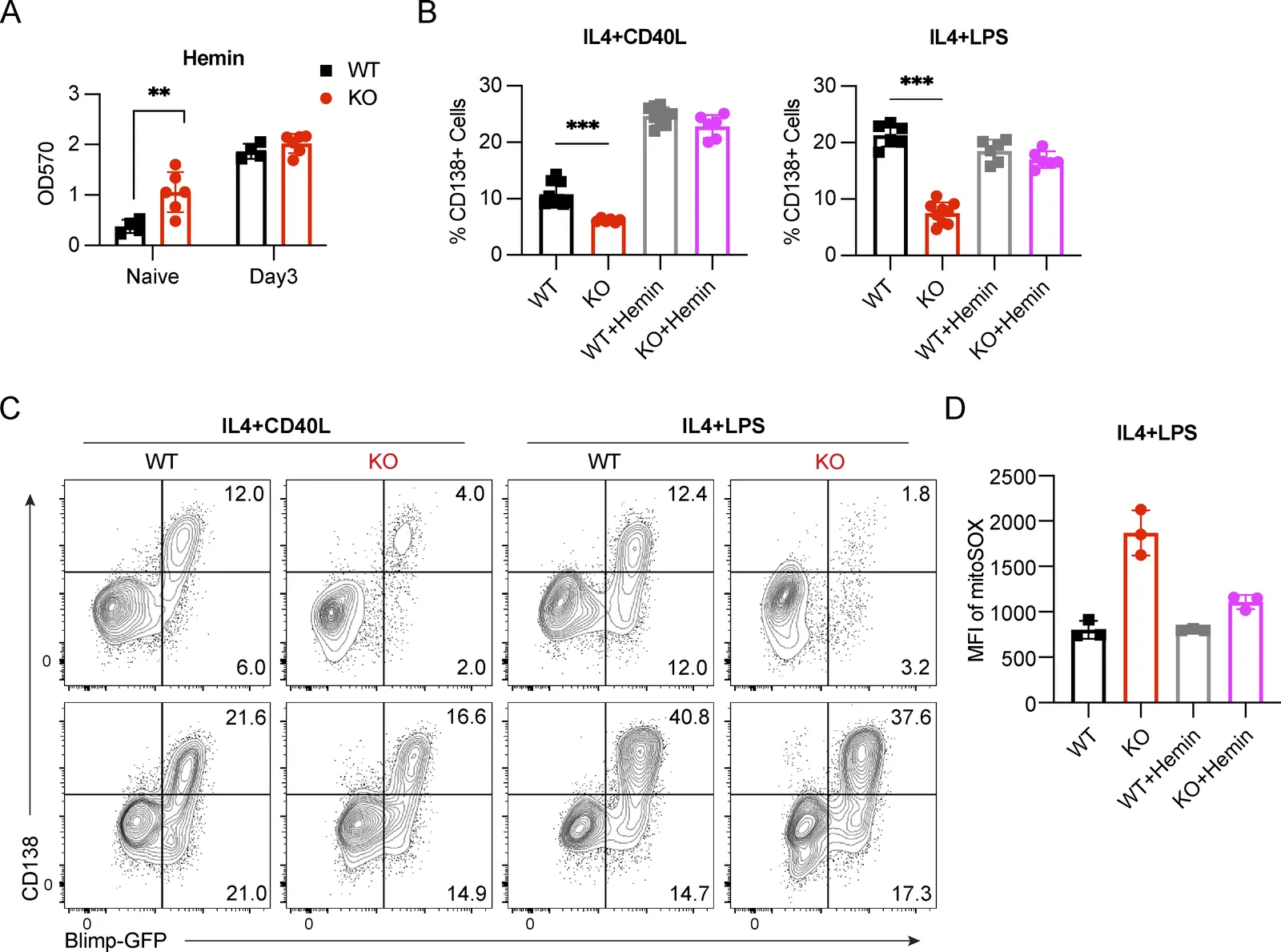
**Hemin rescued plasma differentiation in *Fip200*** ^**−/−**^ **B cells. (A)** Quantification of the hemin level in WT (*n* = 2–3) and *Fip200*^*−/−*^ (*n* = 3–4) B cells at the naïve stage and day 3 after activation. P values were determined by an unpaired *t* test. **P = 0.0072. Three independent experiments were performed, with representative data shown for one. **(B)** Quantification of plasma cell fractions from WT and *Fip200*^*−/−*^ B cells cultured in the presence of IL4+CD40L (left), and IL4+LPS (right) with or without hemin (60 μM) for 3 days. Three independent experiments were performed; data from one experiment are shown (*n* = 1–3 per treatment/genotype). Left: ***P = 0.0004, right: ***P = 0.0004 **(C)** Representative plots of WT and *Fip200*^*−/−*^ B cells expressing Blimp-GFP were stimulated by IL4+CD40L (left) and IL4+LPS (right) without hemin (upper) or with hemin (60 μM, bottom) at day 3 by FACS. Three biological replicates were performed for each treatment; data from one experiment are shown. **(D)** mROS level in WT and *Fip200*^*−/−*^ B cells in the presence of IL4+LPS with or without hemin (60 μM). Two independent experiments were performed with three mice in each group; data from one experiment are shown.

Due to the observed downregulation of heme metabolism in the *Fip200*^*−/−*^ plasma population and to prior work associating insufficient heme synthesis with diminished plasma cell differentiation (Jang et al., 2015), we examined the hemin level in naïve B cells and found it was higher in *Fip200*^*−/−*^ B cells than in WT. 3 days after activation, however, hemin levels increased in both and equalized in terms of total amount relative to their respective starting points—indicating that less hemin was newly generated after activation in KO cells (Fig. 7 A). As hemin enhances the expression of the plasma cell master regulator Blimp-1 and thus affects plasma cell differentiation (Watanabe-Matsui et al., 2011), we investigated whether the addition of hemin could rescue plasma differentiation in *Fip200*^−/−^ B cells. We added 60 µM hemin to the cultures at day 0 and observed increased WT and *Fip200*^−/−^ plasma cell populations at day 3: these increased populations were statistically equivalent between the knockout and WT (Fig. 7 B). We observed similar results in Blimp-1-GFP–expressing B cells treated with hemin (Fig. 7 C). Furthermore, hemin treatment reduced the level of mROS in *Fip200*^*−/−*^ but not in WT B cells, moving it closer to WT (Fig. 7 D). Thus, by bypassing the downregulated heme pathway through supplementation, we reduced the mROS level, reprogrammed B cell fate, and promoted plasma differentiation in *Fip200*^*−/−*^ B cells.

## Discussion

Autophagy plays an important role in B cell development, GC formation, and memory. The investigation of autophagy and the B cell response has been primarily focused on the elongation and maturation of the autophagosome, with little attention paid to the upstream portion of the autophagy pathway. Here, we have found that FIP200, a complex molecule with multiple functions in and beyond the autophagy pathway, regulates the proliferation of B cells after activation and prevents the accumulation of dysfunctional mitochondria, thereby promoting plasma cell differentiation and humoral immune responses.

FIP200’s role in other immune cells varies; for example, downregulation of FIP200 expression in naïve T cells has previously been observed to lead to apoptosis (Xia et al., 2017). Here, however, we found that naïve B cell populations in the spleen were not significantly affected by FIP200 ablation; B-*Fip200*^−/−^ mice showed accumulated pre-B cells and fewer recirculating B cells in the BM, which implied that FIP200 promoted B cell maturation. On activation, in contrast, we observed impaired B cell proliferation in *Fip200*^−/−^ B cells. This is consistent with FIP200’s role in regulating mTOR signaling: Gan et al. found heart and liver cells from *Fip200*^*−/−*^ embryos showed reduced phospho-S6 kinase signal (Gan et al., 2006) and confirmed that FIP200 could interact with the TSC1-TSC2 complex, which regulates the mTOR signaling pathway (Gan et al., 2005). This may in part explain the reduced GC populations we observed in B-*Fip200*^*−/−*^ mice after immunization, as defects in GC formation were previously observed after the ablation of another autophagy component in the B cell compartment, WIPI2 (Martinez-Martin et al., 2017). Although fewer plasma cells were generated in B-*Fip200*^−/−^ mice upon immunization, more IgG class-switched cells were generated and the protein secretion pathway was upregulated, rescuing early-stage NP-specific IgG antibodies (day 11–21). However, as previously observed in ATG5- and ATG7-deficient mice, there were fewer long-lived plasma cells found in FIP200-deficient BM, and a lower boost immune response, supporting a pivotal role of autophagy in long-lived plasma and memory B cell survival (Chen et al., 2014, 2015; Pengo et al., 2013). This combination of decreased plasma differentiation and survival led to a reduction in NP-specific IgG antibodies at later stages (>day 28) after immunization in FIP200-deficient mice. In this respect, FIP200 acts similar to other molecules in the autophagy pathway in the B cell humoral immune response and in the maintenance of plasma and memory B cells.

In macrophages, TLR4 signals are known to activate the NLRP3 inflammasome (Latz et al., 2013; Paik et al., 2021), while CD40L-CD40 signaling inhibits NLRP3 inflammasomes (Guarda et al., 2009). Furthermore, ATG16L1- or ATG7-deficient macrophages show increased caspase-1 activation, indicating that autophagy plays an important role in regulating NLRP3 degradation (Saitoh et al., 2008). Here, we similarly confirmed that B cells activated by LPS+IL4 were prone to form low levels of NLRP3 inflammasomes, which were then cleared in a FIP200-dependent manner. Furthermore, we observed increased cell death in FIP200-deficient B cells after LPS+IL4 stimulation. In contrast to LPS activation, CD40L stimulation *in vitro* or *in vivo* did not induce significant cell death in *Fip200*^*−/−*^ B cells, nor were there significant differences in the mRNA profiles of GC B cells isolated from *B-Fip200*^−/−^ and WT mice. This lack of difference in GC B cells in the 10x data may be attributed to the fact that these populations were the result of a T-dependent immunization, and most GC B cells obtain help from CD4^+^ T cells for proliferation and somatic hypermutation (Cyster and Allen, 2019; De Silva and Klein, 2015); furthermore, GC B cell development is less dependent on the canonical autophagy pathway (Chen et al., 2014; Martinez-Martin et al., 2017; Pengo et al., 2013). Thus, the high rate of cell death upon TLR4 signaling activation is likely due to the accumulation of NLRP3 inflammasomes after the ablation of FIP200; as NLRP3 is detected in splenic plasmablasts but not in the plasma cells in the ImmGen ULI RNA-Seq dataset (Heng et al., 2008), removal of NLRP3 inflammasomes may also be important for plasma differentiation.

Hemin is known to induce heme oxygenase-1 (HO-1) expression, which is critical for antiviral effects on SARS-CoV-2 infection, and antitumor effects on prostate, breast, and colon cancer (Andrés et al., 2014; Coló et al., 2023; Gandini et al., 2019; Gueron et al., 2009; Jaworski et al., 2017; Kim et al., 2021; Kodagoda Gamage et al., 2021). Furthermore, the innate immune system can be trained by heme against sepsis through the activation of spleen tyrosine kinase/c-Jun N-terminal kinase (Jentho et al., 2021). Hemin is also known to regulate B cell fate, and its addition drives both naïve and memory B cells to differentiate into plasma cells (Jang et al., 2015; Price et al., 2021; Tsui et al., 2018; Watanabe-Matsui et al., 2011). While the specific mechanism of action underlying that differentiation is unclear, it has been observed that the reduced form, heme, binds to transcription factor Bach2, diminishing the half-life of Bach2 and inhibiting its DNA binding activity, and in turn enhancing Blimp-1 expression (Watanabe-Matsui et al., 2011). However, *Bach2*^*−/−*^ B cells also show increased plasma differentiation when heme was added (Watanabe-Matsui et al., 2011), suggesting another mechanism may be at play. Jang et al. previously demonstrated that the addition of an antioxidant to B cell cultures increased the heme level in B cells, suggesting that mROS inhibits cellular heme level (Jang et al., 2015). Previous work, however, did not find that the addition of hemin in B cell culture affected the mROS level in WT B cells, though it did reduce mROS in B cells where the *Prkcb*^−/−^, a signaling molecule that propagates NF-κB signaling upon B cell activation, had been ablated (Tsui et al., 2018), which is in line with our observations in *Fip200*^*−/−*^ B cells. Thus, while we hypothesize that mROS and hemin are mutually regulated, the mechanism whereby this occurs will require further investigation.

Mitochondrial mass is distinct in different B cell states: B cells within the GC show increased mitochondrial mass, while the mitochondrial mass in plasma cells is lower (Haniuda et al., 2020; Yazicioglu et al., 2023). Upon LPS+IL4 stimulation, mitochondrial mass increases in B cells at days 1 and 2, then decreases at days 3 and 4. B cells sorted at day 2.5 containing less mitochondrial mass were more prone to maintain or differentiate into plasma (Jang et al., 2015). Previously, our group also observed that WIPI2-KO B cells show diminished mitochondrial mass and lower mitochondrial membrane potential upon CpG stimulation at day 3 and are more likely to differentiate into plasma cells than WT (Martinez-Martin et al., 2017). Here, we found decreased mitochondrial mass at day 3 upon CD40L+IL4 or LPS+IL4 stimulation in WT B cells, as well as a higher rate of mitophagy in WT plasma cells relative to other B cells after activation. FIP200 KO B cells accumulated more mitochondrial mass, had higher mROS, and, in line with those previous observations, underwent plasma differentiation less frequently. Contrary to our expectations, mROS levels in long-lived plasma cells from B-*Fip200*^−/−^ mice were lower than observed in WT. However, as there were also far fewer LLPCs in B-*Fip200*^−/−^ mice, it is possible that mROS levels increased in a runaway fashion in KO cells, leading to cell death and removal from the population; alternatively, FIP200 may ameliorate the effects of high mROS. Recent studies found that FIP200 could mediate an unconventional lysosomal targeting pathway bypassing the lipidation of LC3 (Chen et al., 2014; Huyghe et al., 2022; Ohnstad et al., 2020; Okamoto et al., 2020; Schlütermann et al., 2021; Vargas et al., 2019; Wang et al., 2021), and our observations indicate that TAX1BP1 participates in mitophagy but fails to fuse with the lysosome in the absence of FIP200. Therefore, we propose that FIP200 participates in an LC3 lipidation–independent form of mitophagy mediated by TAX1BP1, the disruption of which inhibits the removal of dysfunctional mitochondria, which is critical for plasma cell differentiation.

In summary, we found that FIP200 plays distinct regulatory roles during B cell activation and differentiation. When B cells are activated through the TLR4 signaling pathway, FIP200 initiates autophagy to remove NLRP3 inflammasomes, contributing to B cell survival. FIP200 also participates in an LC3 lipidation–independent form of mitophagy, and its ablation leads to the accumulation of dysfunctional mitochondrial mass and increased mROS. Furthermore, FIP200 regulates heme metabolism, which may contribute to the plasma differentiation and could be corrected by the application of hemin. By removing dysfunctional mitochondria, FIP200 governs mitochondrial homeostasis and promotes plasma differentiation.

## Materials and methods

### Animal breeding and generation

*Fip200* ^*flox/flox*^ animals were developed by Jun-Lin Guan of the University of Cincinnati, College of Medicine, and were backcrossed to mice expressing Cre recombinase from the *mb1* (CD79a) promoter (Hobeika, 2006). *Mb1* Cre^−^ littermates were used as WT controls. C57BL/6J mice were either obtained internally or purchased from the Jackson Laboratory (IMSR_JAX:000664). *Blimp*-GFP mice (Kallies et al., 2004) were obtained from the internal breeding facility, and these mice were crossed with *Fip200*^*flox/flox*^ mice. MitoQC mice were generated as described in McWilliams et al. (2016), based on the strategy in Allen et al. (2013), by Ian Ganley at the University of Dundee, UK. Briefly, a CAG promoter and ORF for the mCherry-GFP-FIS1 fusion protein were inserted at the mouse Rosa26 locus via recombination-mediated cassette exchange in a C57BL/6 background (TaconicArtemis GmbH); we then crossed these mice with our *Fip200*^*flox/flox*^ mice. Mice used in experiments described here were bred and maintained at the animal facility at the Ragon Institute. All experiments were approved by the Institutional Animal Care and Use Committee of the Massachusetts General Hospital. Animal experiments at the Ragon Institute were conducted in accordance with Association for Assessment and Accreditation of Laboratory Animal Care International regulations.

### Immunization, ELISA, and ELISPOT

Mice of both sexes (8–13 wk old) were immunized intraperitoneally with 30 μg NP_29_-KLH (Biosearch Technologies) in 4 mg Alum (Thermo Fisher Scientific) and boosted with 50 μg NP_29_-KLH in PBS at day 85. Blood samples were collected by submandibular bleeding on days 0, 11, 28, 42, 85, 92, and 99 after immunizations. To detect NP-specific antibody titers by ELISA, 5 μg/ml NP_29_-BSA and NP_7_-BSA was used for capture, and Alkaline Phosphatase AffiniPure Goat Anti-Mouse IgM, µ chain specific (AB_2338537), Anti-Mouse IgG, Fcγ fragment specific (AB_2338535; Jackson ImmunoResearch), biotinylated anti-mouse IgG (AB_2794296; SouthernBiotech), IgG1 (AB_2794413; SouthernBiotech), IgG2b (AB_2794523; SouthernBiotech), IgG2c (AB_2794467; SouthernBiotech), IgG1 (AB_2794413; SouthernBiotech), and IgA (AB_2794374; SouthernBiotech) were used for detection. NP-specific ASCs were detected using multiscreen filter plates (Millipore). Plates were activated with pure ethanol (MilliporeSigma), washed with sterile PBS, and coated overnight with 10 µg/ml NP_29_-BSA and NP_7_-BSA at 4°C. Activated plates were blocked in complete B cell medium for 3 h at room temperature (RT). Serial dilutions of splenocytes collected from immunized mice were plated, and biotinylated anti-mouse IgM (AB_2794242; SouthernBiotech) or IgG was used for detection. Alkaline phosphatase–streptavidin (Sigma-Aldrich) and phosphorylated nitrophenyl phosphate (ELISA, Sigma-Aldrich) or BCIP/NIST (ELISPOT, Sigma-Aldrich) were used to develop ELISA and ELISPOT plates. BioTek Synergy Neo2 Hybrid Multimode Reader plate reader (BioTek) was used to read absorbance at 405 nm. ELISPOT plates were imaged via an ImmunoSpot analyzer (ImmunoSpot).

### Flow cytometry

Single-cell suspensions for flow cytometry experiments were prepared from homogenized spleen, lymph nodes, or BM. Erythrocytes were destroyed with lysis buffer (Lonza). Prior to antibody staining, Fc receptors were blocked using anti-CD16/32 (2.4G2) (AB_394657; BD Bioscience) antibodies and dead cells were labeled with Live/Dead Blue (Thermo Fisher Scientific) for 20 min on ice. Then, cells were centrifuged and resuspended with antibody mix diluted in DPBS with 2% FBS at 1:200 ratio and incubated on ice for 20 min. Next, cells were washed once with DBPS with 2% FBS before flow cytometry. Antibodies used include an applicable combination of the following: B220 (RA3-6B2, AB_893354), TCRβ (H57-597, AB_893624), CD21 (7G6, AB_10924591), CD4 (GK1.5, AB_312699), CD8a (53–6.7, AB_312753), CD3 (145–2C11, AB_1877170), CD23 (B3B4, AB_2563438), CD24 (M1/69, AB_2566730), CD43 (S7, AB_10895376), Ly-51 (6C3, AB_2573797), Sca-1 (D7, AB_313343), Ter-119 (TER-119, AB_2137788), F4/80 (QA17A29, AB_2894638), Gr-1 (RB6-8C5, AB_2137485), CD38 (90, AB_2890672), CD95 (Jo2, AB_396768), CD138 (281-2, AB_10916119), IgG1 (A85.1, AB_394862), CD5 (53–7.3, AB_2563929), CD11b (M1/70, AB_10893803), IgM (II/41, AB_2741424), IgD (11-26c.2a, AB_2562887), CXCR4 (2B11, AB_2737757), CD86 (GL-1, AB_313149), PD-L2 (TY25, AB_2566345), CD45.1 (A20, AB_11124743), CD45.2 (104, AB_493731), CD80 (16-10A1, AB_2075999).

For intracellular staining, cells were fixed and permeabilized with Fixation and Permeabilization Solution (BD Biosciences) for 20 min on ice, then blocked with 3% BSA in Perm/Wash Buffer (BD) for 30 min at room temperature. Next, cells were stained with IRF4 (IRF4.3E4, AB_2564048) and PAX5 (1H9, AB_2562573) (BioLegend) for 30 min at room temperature, then washed twice for flow cytometry. Data were acquired on LSRFortessa (BD Bioscience) and analyzed with FlowJo (TreeStar).

### Cell culture, isolation, labeling, immunoblotting, and hemin detection

B cells were cultured in complete B cell medium (RPMI supplemented with 10% FCS, 25 mM Hepes, GlutaMAX, nonessential amino acids, penicillin–streptomycin (Thermo Fisher Scientific), and 50 μM β-mercaptoethanol (MilliporeSigma)). Naïve B and CD4^+^ T lymphocytes were purified from spleens using negative B cell or CD4^+^ T cell selection, resulting in B and T cells with more than 90% purity (Miltenyi Biotec). As described in Burbage et al. (2014), 10^7^ B cells per ml were labeled with 2 μM CTV (Thermo Fisher Scientific) for 5 min at 37°C and washed with complete B cell medium. Labeled cells were stimulated in complete B cell medium, supplemented with combinations of 10 ng/ml IL4 (R&D Systems) with 5 µg/ml LPS (Sigma-Aldrich), 50 ng/ml CD40L (R&D Systems), or 3 µg/ml CpG (ODN1826). CTV dilution was measured daily by flow cytometry for 3 days. The differentiation of plasmablast and IgG1 populations was detected by CD138 (281-2, AB_10916119) and IgG1 (A85.1, AB_394862), respectively. OT-II T cells were labeled with 5 μM CFSE (Thermo Fisher Scientific), and WT or *Fip200*^*−/−*^ MD4 B cells were labeled with 2 μM CTV (Thermo Fisher Scientific), then intravenously injected into CD45.1 recipient mice. At day 1, recipient mice were given 30 μg HEL-OVA intravenously. CTV or CFSE dilution was measured by flow cytometry at day 4.

For immunoblotting, stimulated cells were collected and lysed in lysis buffer (20 mM Tris-HCl, pH 8.0, 150 mM NaCl, 5 mM EDTA, Protease Inhibitor Cocktail [Roche], 10 mM NaF, 1 mM Na_3_VO_4_, and 1% NP-40) for 30 min on ice, and samples were loaded into 4–20% Mini-PROTEAN TGX Precast Protein Gels (Bio-Rad) for electrophoresis and transferred via electrophoresis to PVDF filters using the Mini-PROTEAN and Trans-Blot system (Bio-Rad). Membranes were blocked by incubation in WB blocking buffer (5% milk in TBS, 0.1% Tween) at RT for 1 h and probed with specific Abs diluted in WB blocking buffer or 5% BSA in TBS-T. Proteins were detected with antibodies against FIP200 (D10D11, AB_2797913), P62 (D1Q5S, AB_2799160), LC3B (D11, AB_2137707), cleaved caspase-3 (Asp175, AB_2341188), NLRP3 (D4D8T, AB_2722591), cleaved caspase-1 (Asp296) (E2G2I, AB_2923067) (Cell Signaling Technology), and TAX1BP1 (14424-1-AP) (AB_2198921; Proteintech) using the secondary HRP-conjugated anti-rabbit or anti-mouse antibodies (AB_2307391, AB_2338506; Jackson ImmunoResearch) or anti-β-actin−peroxidase antibody (AC-15) (AB_262011; MilliporeSigma). Blot densitometry analysis was performed using Fiji (SCR_002285; National Institutes of Health) software.

Hemin was detected with Hemin Assay Kit (Abcam). Briefly, B cells were collected at indicated time points and lysed with lysis buffer for 15 min on ice at 10^6^ cells/ml, and then, 5 μl cell lysis was used as per the kit’s manufacturer’s instructions to evaluate the relative hemin level in B cells.

### Droplet-based single-cell RNA-Seq

B-*Fip200*^*−/−*^ (five mice) and sibling control mice (four mice) were immunized with 50 μg NP_29_-KLH with 4 mg Alum and sacrificed at day 11. Splenocytes were enriched for plasma, GCs, and memory B cells by depleting non-B cells and IgD^hi^ B cells using Miltenyi LS columns. Enriched B cells were then stained with DAPI, B220 (RA3-6B2, AB_893354), IgD (11-26c.2a, AB_2562887), CD138 (281-2, AB_10916119), NP-APC, CD38 (90, AB_2890672), CD95 (Jo2, AB_396768), and TotalSeq-C0450 anti-mouse IgM antibody (AB_2861022), TotalSeq-C1167 anti-mouse IgG1 antibody (AB_2904428), TotalSeq-C1168 anti-mouse IgG2b antibody (AB_2904430), TotalSeq-C0571 anti-mouse IgD antibody (AB_2876729), TotalSeq-C0228 anti-mouse CD183 (CXCR3) antibody (AB_2832454), TotalSeq-C0225 anti-mouse CD196 (CCR6) antibody (AB_2894629), TotalSeq-C0107 anti-mouse CD21/CD35 (CR2/CR1) antibody (AB_2860653), TotalSeq-C0108 anti-mouse CD23 antibody (AB_2860591), TotalSeq-C0212 anti-mouse CD24 antibody (AB_2819784), TotalSeq-C0557 anti-mouse CD38 antibody (AB_2819786), TotalSeq-C0562 anti-mouse CD83 antibody (AB_2860647), TotalSeq-C0200 anti-mouse CD86 antibody (AB_2860608), TotalSeq-C0917 anti-mouse CD95 (Fas) antibody (AB_2860719), TotalSeq-C0810 anti-mouse CD138 (Syndecan-1) antibody (AB_2860696), and TotalSeq-C0914 anti-mouse CD273 (B7-DC, PD-L2) antibody (AB_2860615, BioLegend). Sorted naïve (B220^+^IgD^hi^), plasma (CD138^+^B220^lo^), GC (B220^+^CD95^+^CD38^−^), and memory (B220^+^CD38^+^NP^+^) live cells from each mouse were then mixed. Single-cell RNA-Seq was run using the 10x Chromium (10x Genomics) system, and cDNA libraries were generated according to the manufacturer’s recommendations (Chromium Single-Cell 5′ Reagent Kit v2 Chemistry, Dual Index). Libraries were sequenced via NextSeq 2000 for Illumina sequencing.

### Single-cell RNA-Seq analysis

Sample preprocessing was performed using the Cell Ranger analysis pipeline (v7.0.0) aligning to the mm10-2020-A reference transcriptome. Sample demultiplexing, QC, normalization, and clustering were performed with the R package Seurat v4.3.0 (Hao et al., 2021).

ADT and HTO counts were added to the Seurat object and normalized using a centered log ratio transformation. Samples were demultiplexed using the Seurat function HTODemux. Cells that expressed fewer than 300 genes, fewer than 50 housekeeping genes (list of housekeeping genes adapted from (Tirosh et al., 2016), or more than 8% mitochondrial genes were removed from downstream analysis. This resulted in a Seurat object with 17,731 genes across 11,500 cells.

Normalization was performed with sctransform (Choudhary and Satija, 2022; Hafemeister and Satija, 2019); any variance introduced by *Ighv*, *Iglv*, and *Igkv* genes was removed by regression. Principal component analysis was performed with the Seurat function RunPCA; clustering was performed with Seurat functions FindNeighbors on 40 principal components and FindClusters with the sensitivity set to 0.6. The first 40 principal components were used in Uniform Manifold Approximation and Projection using the Seurat RunUMAP function. Cluster markers were determined using the Seurat function FindAllMarkers.

The Seurat object was subsetted to remove any contaminating clusters of non-B cells, resulting in a Seurat object with 17,731 genes across 10,990 cells, which was analyzed as described above. Differentially expressed genes between WT and FIP200 KO were determined using the FindMarkers function. For pseudobulking, counts were log-normalized (using functions NormalizeData, FindVariableFeatures, and ScaleData) and counts were aggregated by sample using the AverageExpression function. The heatmap of top cluster markers was generated with the DoHeatmap function.

For plasma cell analysis, the Seurat object was subsetted to only include plasma cell clusters, resulting in a Seurat object with 17,731 genes across 3,249 cells, which was analyzed as described above. IMGT/HighV-QUEST (https://www.imgt.org/IMGTindex/IMGTHighV-QUEST.php) was used to determine the number of mutations. Cells without BCR sequence information were removed from the analysis. Heavy chain mutational load was visualized using the Seurat function VlnPlot. Signature scores for MSigDB (Liberzon et al., 2011, 2015; Subramanian et al., 2005) hallmark gene sets (using dataset c2.cp.kegg.v7.1.symbols.gmt) were calculated using the Vision package v3.0.0 (DeTomaso et al., 2019) as described previously (Ringel et al., 2020).

### Imaging flow cytometry

Purified MitoQC-B cells were activated for 24 h and treated with DMSO or FCCP (5 mM) for 30 min. Isolated naïve or stimulated FIP200-B-KO-MitoQC and FIP200-B-WT-MitoQC-B cells were collected at days 1, 2, and 3. Cells were then blocked with Fc blocker (2.4G2) (BD Bioscience, AB_394657) antibodies together with Live/Dead Violet (Thermo Fisher Scientific) for 20 min on ice. CD138 (281-2, AB_10962911) antibody was used to stain the plasmablast population. Cells were fixed with 2% paraformaldehyde and then suspended in 50 μl PBS.

The ImageStreamX (Amnis) two-camera system with 405-, 488-, 561-, and 642-nm lasers (along with a 785-nm laser for dark-field SSC) was used for imaging flow cytometry. Images (Live/Dead Violet in channel 7, GFP in channel 2, mCherry in channel 4, APC-CD138 in channel 11, and bright field in either channel 1 or 9) were acquired for each cell at 40× magnification, and ∼20,000 cells were analyzed for each experiment. The integrated software INSPIRE (Amnis) was used for data collection, and analysis was performed on the compensated image files using algorithms in IDEAS (Amnis) image analysis software. All single and focused cells used in our analysis had zero saturated pixels. The BDS of live cells expressing both the GFP and mCherry features was used to measure colocalization based on overlapping pixel intensities for GFP and mCherry bright details, using the default MC mask, a union of the pixels from all the channel masks. Example plots and gates are in Fig. S3 B.

### Extracellular flux assay

Naïve and activated B cells were resuspended in Seahorse medium supplemented with 11 mM glucose and 2 mM pyruvate and adjusted to pH 7.4. Cells were settled on a 96-well assay plate (Agilent) coated with polylysine (MilliporeSigma). The Seahorse XF Cell Mito stress test kit was used, and data were recorded with the XF96 Extracellular Flux Analyzer. Resting ECAR was recorded simultaneously.

### MitoTracker, mROS staining

Mitochondrial mass was measured by incubating cells in complete medium with MitoTracker Deep Red (Thermo Fisher Scientific) at recommended concentrations at 37°C and 5% CO_2_ for 30 min. Cells were washed with 2% FBS-supplemented PBS and analyzed by flow cytometry. mROS was measured by incubating cells in HBSS with calcium and magnesium with 500 nM MitoSOX Red for 30 min at 37°C and 5% CO_2_. Cells were washed with HBSS with calcium and magnesium and analyzed by flow cytometry. Data were acquired on LSRFortessa (BD Bioscience) and analyzed with FlowJo (SCR_008520; TreeStar).

### Microscope imaging

B cells isolated from MitoQC-Fip200^f/f^ Mb1-Cre^−/−^ or MitoQC-Fip200^f/f^ Mb1-Cre^+/−^ mice using a Pan B cell isolation kit (Miltenyi) were activated by IL4+CD40L for 2 days. Then, cells were washed with PBS, loaded to poly-L-lysine (Sigma-Aldrich)–treated µ-Slide 8 Well high Glass Bottom (ibidi) chamber, and incubated at 37°C and 5% CO_2_ for 15 min. After that, cells were fixed and permeabilized with BD Cytofix/Cytoperm solution for 30 min at RT. Then, cells were blocked with 5% BSA-supplemented PHEM buffer and stained with anti-TAX1BP1 polyclonal antibody (AB_2198921; Proteintech) at 1:100 dilution. After being washed four times with 500 μl PHEM buffer, cells were stained with Alexa Fluor 647–conjugated goat anti-rabbit IgG antibody (AB_2535813) at 1:1,000 dilution for 1 h at RT, then washed five times with 500 μl PHEM buffer. Cells were imaged by Celldiscoverer 7 with LSM 900 confocal, using Plan-Apochromat 50×/1.2 W objective lens (Zeiss). Images were captured with 488-, 561-, and 647-nm lasers and Airyscan in ZEN (SCR_018163; Zeiss). Images were exported in 16-bit and further processed by Fiji (SCR_002285).

### Experimental data and statistical analysis

Sample sizes were selected as per the literature on similar phenotypes/defects. Where applicable, study group data were compared using paired or unpaired *t* tests; normal distribution was assumed from the literature. Unless stated otherwise, all figures are representative of at least two independent experiments with three or more animals in each group. Neither investigator blinding nor cohort randomization was used. Any data point with an absolute difference from the mean greater than three times the standard deviation was excluded.

All statistical analyses were performed using Prism 10 (SCR_000306; GraphPad, Inc.).

### Online supplemental material

Fig. S1 shows the B cell development in B-*Fip200*^*−/−*^ mice developed for this study; it is related to Fig. 1. Fig. S2 shows changes in *Fip200*^*−/−*^ B cell proliferation, differentiation, and mitochondrial function and mass after IL4+CD40L stimulation and is related to Figs. 3 and 4. Fig. S3 shows the role of TAX1BP1 in mitophagy in MitoQC-B-*Fip200*^*−/−*^ after CD40L stimulation. Fig. S4 shows 10x single-cell RNA-Seq sample preparation and cluster definition. Fig. S5 shows single-cell RNA-Seq of different B cell populations from WT and B-*Fip200*^*−/−*^ mice, related to Fig. 6.

## Supplementary Material

SourceData F3 is the source file for Fig. 3.

SourceData F4 is the source file for Fig. 4.

SourceData FS1 is the source file for Fig. S1.

SourceData FS2 is the source file for Fig. S2.

SourceData FS3 is the source file for Fig. S3.

## Data Availability

Model animals are available from the corresponding author (F.D. Batista) or via reference to acknowledged providers on request, subject to Standard Material Transfer Agreements. RNA-Seq data are available on ArrayExpress (E-MTAB-16123).
