# Supplementary figures and images for: FIP200 regulates plasma B cell differentiation via mitochondrial and heme homeostasis
Source: J Exp Med. 2025 Dec 17;223(3):e20250535. doi: 10.1084/jem.20250535 (PMC12710606; doi:10.1084/jem.20250535)

# Uncropped blots\_Figure 3

## Panel A

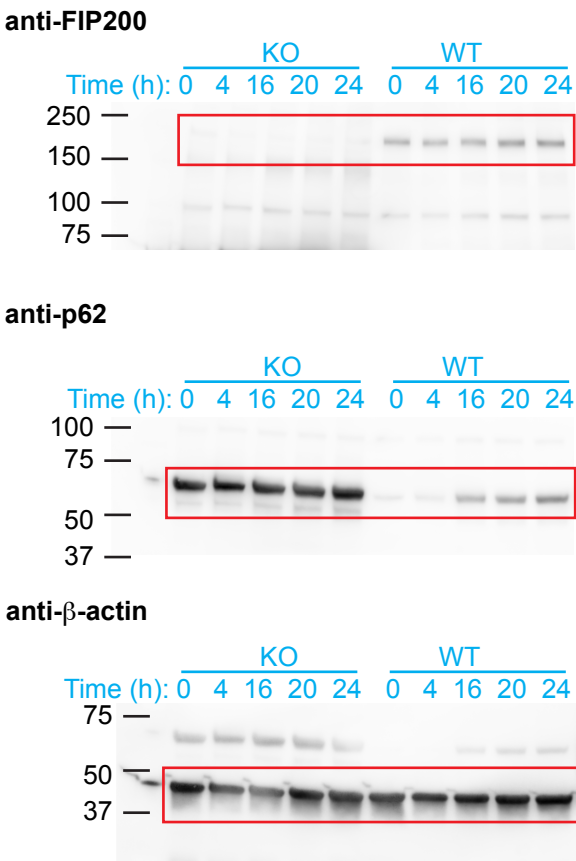

## Panel B

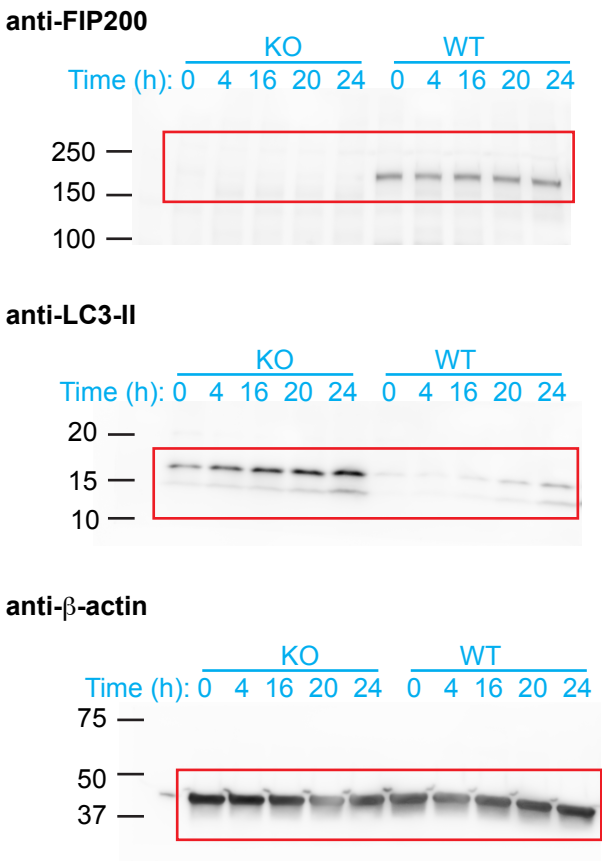

## Panel J

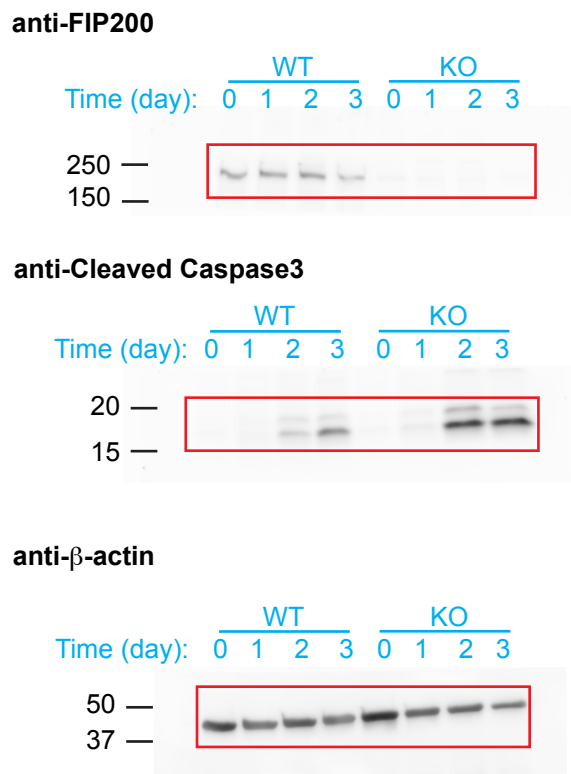

Supplement: SourceData F3 — is the source file for Fig. 3. [file jem_20250535_sourcedataf3.pdf]

# Uncropped blots\_Figure 4

## Panel H

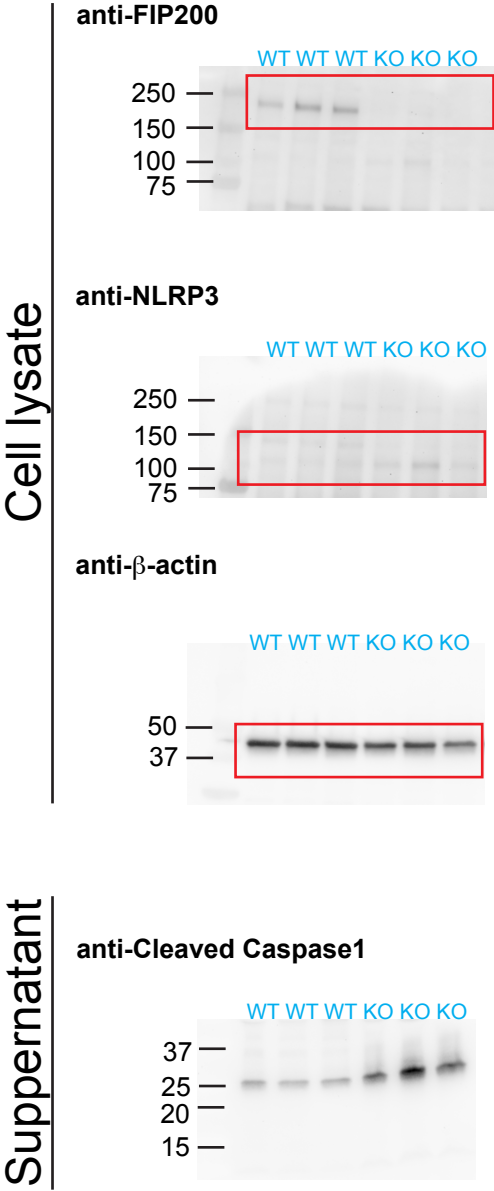

Supplement: SourceData F4 — is the source file for Fig. 4. [file jem_20250535_sourcedataf4.pdf]

# Uncropped blots\_Supplementary Figure 1

## Panel A

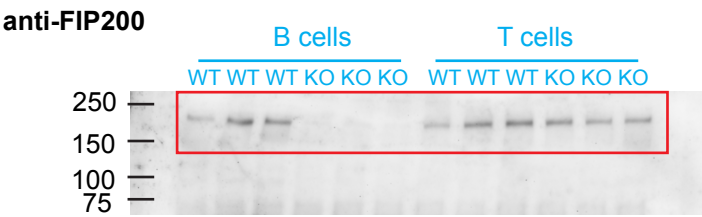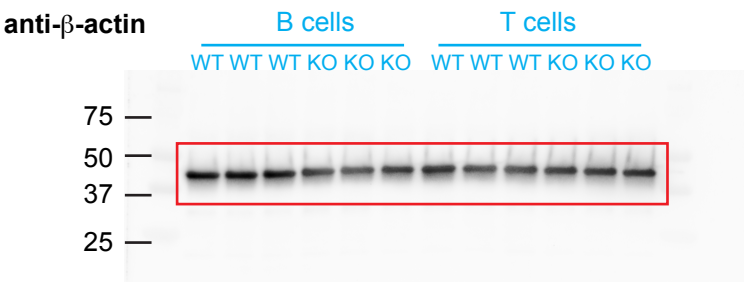

Supplement: SourceData FS1 — is the source file for Fig. S1. [file jem_20250535_sourcedatafs1.pdf]

# Uncropped blots\_supplementary Figure 2

## Panel G

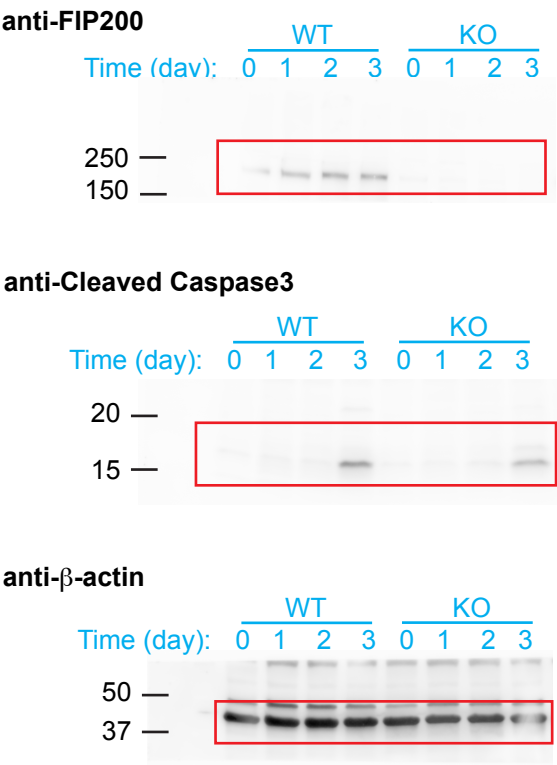

Supplement: SourceData FS2 — is the source file for Fig. S2. [file jem_20250535_sourcedatafs2.pdf]

# Uncropped blots\_Supplementary Figure 3

## Panel E

anti-FIP200

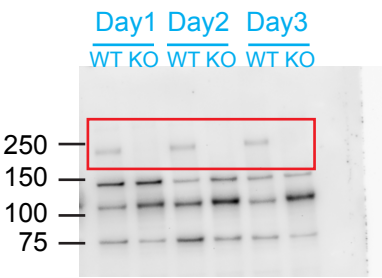

anti-TAX1BP1

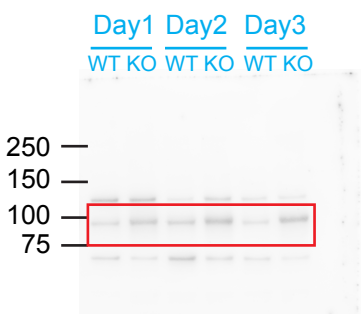

anti-β-actin

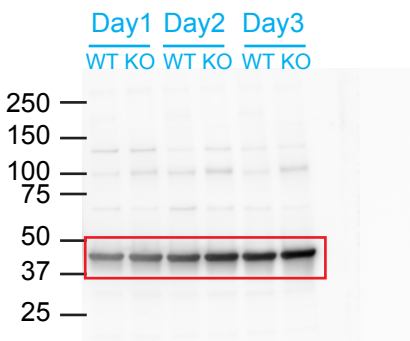

Supplement: SourceData FS3 — is the source file for Fig. S3. [file jem_20250535_sourcedatafs3.pdf]
